# Resolution enhancement with a task-assisted GAN to guide optical nanoscopy image analysis and acquisition

**DOI:** 10.1038/s42256-023-00689-3

**Published:** 2023-07-27

**Authors:** Catherine Bouchard, Theresa Wiesner, Andréanne Deschênes, Anthony Bilodeau, Benoît Turcotte, Christian Gagné, Flavie Lavoie-Cardinal

**Affiliations:** 1 Institute Intelligence and Data (IID), Université Laval, Quebec City, Quebec Canada; 2 CERVO Brain Research Center, Quebec City, Quebec Canada; 3 Département de génie électrique et de génie informatique, Université Laval, Quebec City, Quebec Canada; 4 Département de psychiatrie et de neurosciences, Université Laval, Quebec City, Quebec Canada

**Keywords:** Super-resolution microscopy, Machine learning, Image processing, Cellular neuroscience

## Abstract

Super-resolution fluorescence microscopy methods enable the characterization of nanostructures in living and fixed biological tissues. However, they require the adjustment of multiple imaging parameters while attempting to satisfy conflicting objectives, such as maximizing spatial and temporal resolution while minimizing light exposure. To overcome the limitations imposed by these trade-offs, post-acquisition algorithmic approaches have been proposed for resolution enhancement and image-quality improvement. Here we introduce the task-assisted generative adversarial network (TA-GAN), which incorporates an auxiliary task (for example, segmentation, localization) closely related to the observed biological nanostructure characterization. We evaluate how the TA-GAN improves generative accuracy over unassisted methods, using images acquired with different modalities such as confocal, bright-field, stimulated emission depletion and structured illumination microscopy. The TA-GAN is incorporated directly into the acquisition pipeline of the microscope to predict the nanometric content of the field of view without requiring the acquisition of a super-resolved image. This information is used to automatically select the imaging modality and regions of interest, optimizing the acquisition sequence by reducing light exposure. Data-driven microscopy methods like the TA-GAN will enable the observation of dynamic molecular processes with spatial and temporal resolutions that surpass the limits currently imposed by the trade-offs constraining super-resolution microscopy.

## Abstract (web summary)

Algorithmic super-resolution in the context of fluorescence microscopy is challenging due to the difficulty to reliably represent biological nanostructures in synthetically generated images. Bouchard and colleagues propose a deep learning model for live-cell imaging that can leverage auxiliary microscopy imaging tasks to guide and enhance reconstruction, while preserving the biological features of interest.

## Main

The development of super-resolution optical microscopy (optical nanoscopy) techniques to study the nanoscale organization of biological structures has transformed our understanding of cellular and molecular processes^1^. Such techniques, including stimulated emission depletion (STED) microscopy^2^, are compatible with live-cell imaging, enabling the monitoring of subcellular dynamics with unprecedented spatio-temporal precision. In the design of optical nanoscopy experiments, multiple and often conflicting objectives (for example, spatial resolution, acquisition speed, light exposure and signal-to-noise ratio) must be considered^3,4^. Machine learning-assisted microscopy approaches have been proposed to improve the acquisition processes, mostly by limiting light exposure^3,5,6^. In parallel, several supervised^7–10^ and weakly supervised^11–13^ deep learning approaches have been developed for high-throughput analysis of microscopy images. Deep learning-based super-resolution^5^^,^^14–18^ and domain adaptation^19^ approaches have also been proposed recently for optical microscopy, but concerns and scepticism arise regarding their applicability to characterize biological structures at the nanoscale^20–22^.

Optical nanoscopy techniques exploit the ability to modulate the emission properties of fluorescent molecules to overcome the diffraction limit of light microscopy^23^. In this context, it is challenging to rely on algorithmic methods to generate images of subdiffraction structures that are not optically resolved in the original image^20^. Methods that are optimized for generating images that appear to belong to the target higher-resolution domain do not specifically guarantee that the biological features of interest are accurately generated^22^. Yet, the possibility to super-resolve microscopy images post-acquisition would favourably alleviate some of the compromises between the acquisition parameters in optical nanoscopy^16,24^.

Among the methods developed for algorithmic super-resolution, conditional generative adversarial networks (cGAN)^25^ generate data instances based on a different input value, capturing some of its features to guide the creation of a new instance that fits the target domain. However, the realism of the synthetic images does not ensure that the images are usable for further field-specific analysis, which is limiting their use in optical microscopy. The primary goal for generating super-resolved microscopy images is to produce reliable nanoscale information on the biological structures of interest. Optimizing a network using auxiliary tasks, or multi-task learning, can guide the generator to resolve content that matters for the current context^26^. Various applications of cGANs for image-to-image translation use auxiliary tasks such as semantic segmentation^27,28^, attributes segmentation^29^ or foreground segmentation^30^ to provide spatial guidance to the generator. We adapt this idea in the context of microscopy, where structure-specific annotations can direct the attention to subtle features that are only recognizable by trained experts.

We propose to guide the image-generation process using an auxiliary task that is closely related to the biological question at hand. This approach improves the applicability of algorithmic super-resolution and ensures that the generated features in synthetic images are consistent with the observed biological structures in real nanoscopy images. Microscopy image analysis tasks that are already routinely solved with deep learning^9^ (for example, segmentation, detection and classification) can guide a cGAN to preserve the biological features of interest in the generated synthetic images. Here we introduce a task-assisted GAN (TA-GAN) for resolution-enhanced microscopy image generation. The TA-GAN relies on an auxiliary task associated with structures that are unresolved by the input low-resolution modalities (for example, confocal or bright-field microscopy) but are easily distinguishable in the targeted super-resolution modalities (for example STED or structured illumination microscopy (SIM)). We expand the applicability of the method with a variation called TA-CycleGAN, based on the CycleGAN model^31^, applicable to unpaired datasets. Here the TA-CycleGAN is applied to domain adaptation for STED microscopy of fixed and living neurons. Our results show that the TA-GAN and TA-CycleGAN models improve the synthetic representation of biological nanostructures compared with other algorithmic super-resolution approaches. Specifically, our method is useful to (1) guide the quantitative analysis of nanostructures, (2) generate synthetic datasets of different modalities for data augmentation or to reduce the annotation burden and (3) predict regions of interest for machine learning-assisted live-cell STED imaging.

### Results

#### Task-assisted super-resolution image generation

**Fig. 1:**
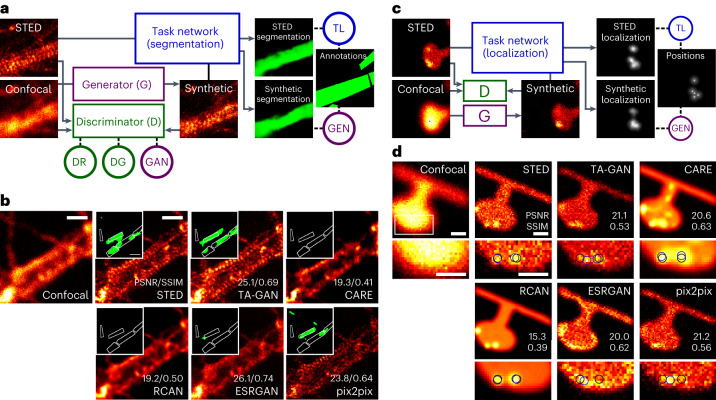
The TA-GAN method. **a**, Architecture of the TA-GAN_Ax_. The losses (circles) are backpropagated to the networks of the same colour: the generator (violet), the discriminator (green) and the task network (blue). DG, discriminator loss for generated images; GEN, generation loss; GAN, GAN loss; DR, discriminator loss for real images; TL, task loss. The TA-GAN_Ax_ is applied to the axonal F-actin dataset using the segmentation of F-actin rings as an auxiliary task to optimize the generator. **b**, Representative example chosen out of 52 test images for the comparison of the TA-GAN_Ax_ and algorithmic super-resolution baselines on the axonal F-actin dataset. The confocal image is the low-resolution input and the STED image is the aimed ground truth. Insets: segmentation of the axonal F-actin rings (green) predicted by the U-Net_fixed-ax_ with the bounding boxes (white line) corresponding to the manual expert annotations^13^. PSNR and SSIM metrics are written on the generated images. Scale bars, 1 μm. **c**, The TA-GAN_Nano_ is trained on the simulated nanodomain dataset using the localization of nanodomains as the auxiliary task. **d**, Representative example chosen out of 75 test images for the comparison of the TA-GAN_Nano_ with the baselines for nanodomain localization. The black circles represent the position of the nanodomains on the ground-truth datamap and the blue circles represent the nanodomains identified by an expert on images from the test set (Methods). The intensity scale is normalized for each image by its respective minimum and maximum values. Scale bars, 250 nm.

Deep learning methods designed for synthetic microscopy image generation have been shown to be effective for deblurring and denoising confocal images^15,16,18^. To increase the accuracy of resolution enhancement approaches applied to the generation of complex nanoassemblies, we consider the combination of a cGAN with an additional convolutional neural network, the task network (Fig. 1a), targeting an image analysis task relevant to the biological structures of interest. Three individual networks form the TA-GAN model: (1) the generator, (2) the discriminator and (3) the task network (Fig. 1a). The chosen auxiliary task should be achievable using the high-resolution modality only, ensuring that it is informative about content that is not resolved in the low-resolution input modality. The error between the task network predictions and the ground-truth annotations is backpropagated to the generator to optimize its parameters (Methods). The TA-GAN is trained using pairs of low-resolution (confocal or bright field) and super-resolution (STED or SIM) images.

**Extended Data Fig. 1:**
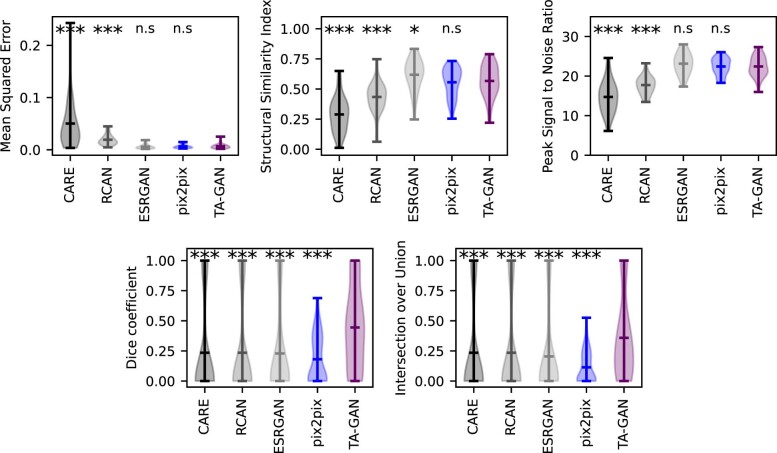
Generation accuracy of TA-GANAx. compared with resolution enhancement baselines. Comparison of TA-GAN_*Ax*._ with the resolution enhancement baselines using three image evaluation metrics : 1) Mean squared error (MSE), 2) Structural Similarity Index Measure (SSIM), 3) Peak Signal to Noise Ratio (PSNR), and two segmentation evaluation metrics : 1) Dice Coefficient (DC), 2) Intersection over Union (IOU). For the image metrics, images are normalized to 0-1 using min-max normalization. The segmentation predictions are computed with the U-Net_*fixed*−*ax*._ on the synthetic images generated with each approach. Metrics are computed using the real STED image and its segmentation by U-Net_*fixed*−*ax*._ as the reference. The score for DC and IOU is 1 if both the reference and prediction are empty. The performance of the TA-GAN is significantly better than all baseline for both segmentation metrics. For the image similarity metrics, TA-GAN performs significantly better than CARE and RCAN, and is similar to ESRGAN and pix2pix. Statistical analysis: Mann-Whitney U test^62^ for the two-sided hypothesis that the distribution underlying the results for each baseline is the same as the distribution underlying the TA-GAN results. Violin plots show the minimum, maximum and mean of each distribution.(*** *p* < 0.001, n.s. *p* > 0.05). n=52 independent images. Source data

The first TA-GAN model, TA-GAN_Ax_, is trained on the axonal F-actin dataset^13^ to generate STED images of the axonal F-actin lattice from confocal images (Fig. 1b). The auxiliary task identified to train the TA-GAN_Ax_ is the segmentation of the axonal F-actin rings, which cannot be resolved with confocal microscopy^32^ (Fig. 1a). The segmentation network output is used to compute the generation loss and to evaluate the generation performance at the test time. The image super-resolution baselines content-aware image restoration (CARE)^16^, residual channel attention network (RCAN)^15^, enhanced super-resolution generative adversarial networks (ESRGAN)^33,34^ and pix2pix^35^ are trained on the axonal F-actin dataset and applied to the generation of a synthetic resolution-enhanced image from an input confocal image (Fig. 1b, first row). We additionally evaluate the performance of the image denoising baselines denoising convolutional neural network (DnCNN)^36,37^ and Noise2Noise^37,38^ on the confocal-to-STED image translation task (Supplementary Fig. 1). Comparison between the results of the TA-GAN_Ax_ with the baselines reveals that the pixel-wise mean square error (MSE), structural similarity index (SSIM) and peak signal-to-noise ratio (PSNR) between generated and ground-truth STED images are either improved or similar using the TA-GAN_Ax_ (Extended Data Fig. 1 and Supplementary Figs. 2 and 3). To evaluate the accuracy of each baseline in the generation of the nanostructure of interest, we evaluate the ability of an independent deep learning model trained on real STED images only^13^, which we refer to as U-Net_fixed-ax_, to segment the F-actin rings in the synthetic images over a held-out subset of the dataset that was not used for training the TA-GAN_Ax_. The TA-GAN_Ax_ model uses the segmentation loss to optimize the generator’s weights, which forces the generated F-actin nanostructures to be realistic enough to be recognized by the task network during training, and by U-Net_fixed-ax_ during testing. The U-Net_fixed-ax_ is applied to the synthetic and real STED images, and the similarity between the resulting pairs of segmentation maps is computed using the Dice coefficient (DC) and intersection over union (IOU) metrics. The improvement in similarity is significant for TA-GAN_Ax_ compared with all baselines (Extended Data Fig. 1).

We created a dataset of nanodomains in simulated shapes of dendritic spines using the pySTED simulation platform^39^ to characterize the conditions where the TA-GAN outperforms the baselines in a controlled environment. The task used to train the TA-GAN for synaptic nanodomain generation (TA-GAN_Nano_) is the localization of the centres of the simulated nanodomains (Fig. 1c). We compare the generated images with the ground-truth datamaps for two analysis tasks: (1) the localization of two nanodomains that are spaced by less than 100 nm, which is too close to be resolved with a standard deconvolution approach (Richardson Lucy^40^), and (2) the counting of nanodomains (2 to 6) separated by variable distances. The localization of the nanodomains can be performed using the TA-GAN synthetic images with similar accuracy to the one obtained using the simulated STED images from the pySTED platform (Supplementary Fig. 4a). For the counting task, the images generated by the TA-GAN, RCAN and pix2pix allow to count up to six nanodomains that cannot be resolved in the simulated confocal images within a simulated spine (Supplementary Fig. 4b). Similarly to the results obtained on the axonal F-actin dataset, TA-GAN and pix2pix are the two algorithmic super-resolution approaches that generate synthetic images with the highest similarity to the target domain for the simulated nanodomain dataset, preserving image features such as the signal-to-noise ratio, background level and spatial resolution (Fig. 1d and Supplementary Fig. 5).

**Fig. 2:**
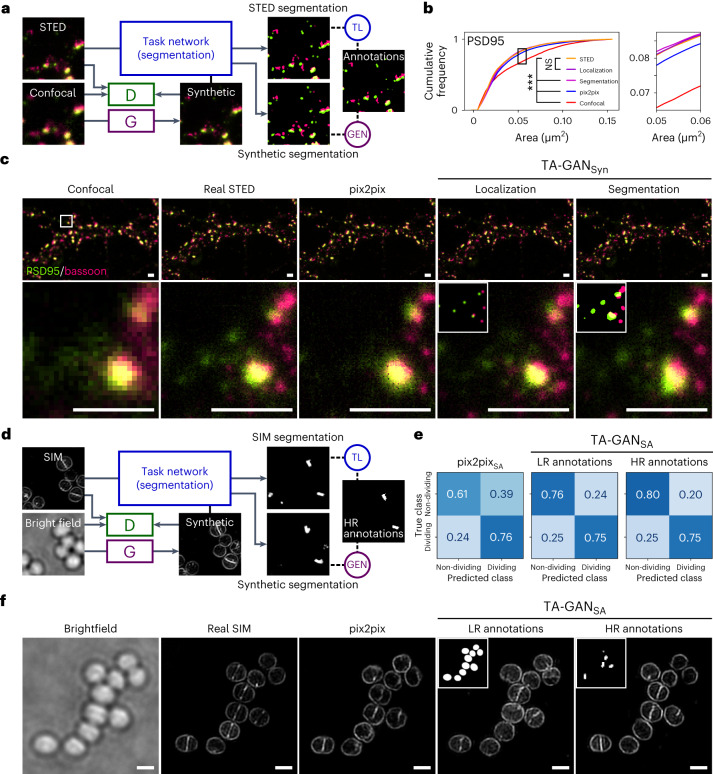
Dataset-specific tasks drive reliable resolution enhancement with the TA-GAN approach. **a**, Two TA-GAN models designed for the synaptic protein dataset are trained using one of two auxiliary tasks: the segmentation of the protein clusters (shown) or the localization of the weighted centroids (Supplementary Fig. 6). **b**, Comparison between the different approaches for the characterization of synaptic cluster morphological features. Shown is the cumulative distribution of the cluster area for PSD95 (see Supplementary Fig. 7 for other features). Statistical analysis: two-sided two-sample Kolmogorov–Smirnov test for the null hypothesis that the continuous distribution underlying the results for each baseline is the same as the one underlying the STED results (***P < 0.001, not significant (NS) *P* > 0.05). **c**, Representative crop chosen from one of the nine test images for the generation of synthetic two-colour images of PSD95 and bassoon using the non-task-assisted baseline (pix2pix), the TA-GAN_Syn_ with the localization task and the TA-GAN_Syn_ with the segmentation task. Insets: localization and segmentation annotations used to train the two TA-GAN_Syn_ models. Scale bars, 1 μm. Each crop is normalized to the 98th percentile of its pixel values for better visualization of dim clusters. **d**, The TA-GAN_SA_ models designed for the *S. aureus* dataset are trained using a segmentation task with annotations requiring only the LR bright-field image or annotations requiring the HR SIM image. **e**, Confusion matrices for the classification of dividing and non-dividing cells on the test set of the *S. aureus* dataset (*n* = 410 cells in five images). The TA-GAN_SA_ trained with HR annotations achieves better performance in generating the boundaries between dividing bacterial cell, a morphological feature visible only with SIM microscopy, compared with pix2pix and the TA-GAN_SA_ trained with LR annotations. **f**, Representative crop chosen from one of the five test images of the *S. aureus* dataset generated with pix2pix and the TA-GAN_SA_ trained with LR and HR annotations. Insets: LR and HR annotations used to train the two TA-GAN_SA_ models. Scale bars, 1 μm. Source data

The TA-GAN model requires the definition of a task that steers the training of the generator towards the accurate extraction of subresolution information. The addition of this task is what differentiates TA-GAN from baselines such as pix2pix. We therefore evaluate how the choice of task impacts the performance using two different datasets. For the synaptic proteins dataset^41^, we evaluate the approach using a localization (Fig. 2a) and a segmentation task (Supplementary Fig. 6). The annotations are automatically generated using the pySODA analysis strategy^41^. For the localization task, we use the weighted centroids of the clusters, whereas for the segmentation task the masks are generated with wavelet segmentation^42^. We show that both tasks can be used to guide the synthetic image generation (Fig. 2b,c), but that the localization task allows to generate synaptic protein clusters with morphological features that are more similar to the one observed in the real images (Supplementary Figs. 7 and 8).

We evaluate how the precision of the labels used for the task impacts the generation accuracy using the publicly available dataset of *Staphylococcus aureus* cells from DeepBacs^43,44^. *S. aureus* bacteria are very small (around 1 μm diameter), and monitoring their morphology changes and cell division processes requires subdiffraction resolution^45^. The TA-GAN_SA_ is trained for bright-field-to-SIM resolution enhancement using a classification task based either on: (1) low-resolution (LR) annotations generated from the bright-field modality (Supplementary Fig. 6) or (2) high-resolution (HR) annotations of dividing cell boundaries obtained from the SIM modality (Fig. 2d). We evaluate how the images generated with the algorithmic super-resolution approaches can be used for the classification of dividing and non-dividing bacterial cells, a task that is not achievable using only bright-field microscopy images (Supplementary Fig. 9). Training the TA-GAN_SA_ model using the HR annotations leads to an improved classification performance combined with improved realism of the synthetic images (Fig. 2e,f).

#### Domain adaptation on unpaired datasets

**Fig. 3:**
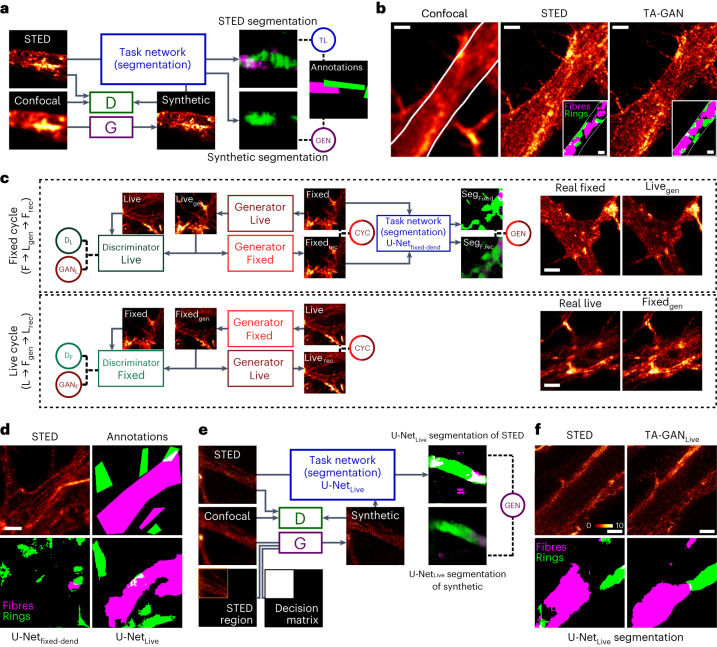
Domain adaptation. **a**, The semantic segmentation of F-actin rings (green) and fibres (magenta) is used as the auxiliary task to train the TA-GAN_Dend_. **b**, Example of confocal, real STED and TA-GAN_Dend_ synthetic images chosen among 26 test images. Insets: the regions identified as rings and fibres by the U-Net_fixed-dend_ trained on real STED images^13^. White solid line shows the border of the dendritic mask generated from the MAP2 channel, following the methods presented in ref. ^13^. **c**, The same semantic segmentation task is used to train the TA-CycleGAN. The reference to compute the TL is the segmentation of real fixed-cell STED images by U-Net_fixed-dend_. The fixed cycle (top) uses U-Net_fixed-dend_ to encourage semantic consistency between the input fixed-cell image and the end-of-cycle reconstructed image. The live cycle (bottom) does not use a task network, enabling the use of non-annotated images from the live F-actin dataset. Once trained, the TA-CycleGAN can generate domain-adapted datasets (right). D_L_, discriminator loss for live-cell images; D_F_, discriminator loss for fixed cell images; GAN_L_, GAN loss for live-cell images; GAN_F_, GAN loss for fixed cell images; CYC, cycle loss; GEN, generation loss; L_rec_, live reconstructed; L_gen_, live generated; F_rec_, fixed reconstructed; F_gen_, fixed generated; Live_gen_, generated live-cell image; Fixed_gen_, generated fixed cell image. **d**, Representative example chosen among 28 annotated live-cell STED test images for the segmentation of F-actin nanostructures. The nanostructures on the live-cell STED images (top left) are not properly segmented by the U-Net_fixed-dend_ (bottom left). The U-Net_Live_ is trained with synthetic images generated by the TA-CycleGAN to segment the F-actin nanostructures on real live-cell STED images. The segmentation predictions generated by the U-Net_Live_ (bottom right) are similar to the manual expert annotations (top right). **e**, The semantic segmentation task is used to train the TA-GAN_Live_. The generator of the TA-GAN_Live_ takes as input the confocal image as well as an STED subregion and a decision matrix indicating the position of the STED subregion in the FOV (Methods). **f**, Representative example of real and synthetic live-cell STED images of F-actin generated with TA-GAN_Live_, chosen among the initial images from 159 imaging sequences. The annotations of both real and synthetic images are obtained with the U-Net_Live_. Colour bar: raw photon counts. Scale bars, 1 μm.

For many microscopy modalities, paired and labelled training datasets are not directly available, or would require a high annotation burden from highly qualified experts. On the basis of the results obtained using confocal and STED image pairs on fixed neurons, we wanted to expand the applicability of the TA-GAN to unpaired datasets—here, images of fixed and living cells. We first validate that the TA-GAN can be applied to the dendritic F-actin dataset^13^ using the semantic segmentation of F-actin rings and fibres in dendrites of fixed neurons (Fig. 3a). The trained TA-GAN_Dend_ generates synthetic nanostructures that are successfully segmented by the U-Net_fixed-dend_, which recognizes dendritic F-actin rings and fibres in real STED images^13^ (Fig. 3b). Similar to results previously obtained from real STED images^13^, the segmentation of the synthetic images with U-Net_fixed-dend_ shows that the area of the F-actin rings significantly decreases as the neuronal activity increases, whereas the opposite is observed for F-actin fibres (Supplementary Fig. 10). Using our task-assisted strategy, we next trained a CycleGAN^35^ model, as it was precisely developed for image domain translation on unpaired datasets. The TA-CycleGAN can be applied to the translation between two microscopy modalities or experimental conditions in which the same biological structure can be observed (here the F-actin cytoskeleton in cultured live and fixed neurons) without the need for paired images. To this aim we generated the live F-actin dataset consisting of confocal and STED images of F-actin nanostructures in living neurons using the far-red fluorogenic dye SiR-actin^46^.

The TA-CycleGAN includes two generators that are trained to first perform a complete cycle between the two domains (fixed- and live-cell STED imaging), and then to compare the ground-truth input image with the generated end-of-cycle image (Fig. 3c). In the generic CycleGAN model, the losses are minimized when the generated images appear to belong to the target domain and the MSE between the input and output is minimized. In TA-CycleGAN we add a task network, here the U-Net_fixed-dend_, which performs the semantic segmentation of dendritic rings and fibres. The U-Net_fixed-dend_ is applied to the real fixed STED images and the end-of-cycle reconstructed fixed STED images (Fig. 3c). The generation loss is computed as the MSE between these segmentation masks. At inference, the trained TA-CycleGAN translates images of a given structure (here F-actin) but with image features (for example, spatial resolution, signal-to-noise ratio, background level) corresponding to the target domain (here live-cell imaging). The translated F-actin dataset was generated by applying the TA-CycleGAN to the dendritic F-actin dataset (Supplementary Fig. 11).

**Extended Data Fig. 2:**
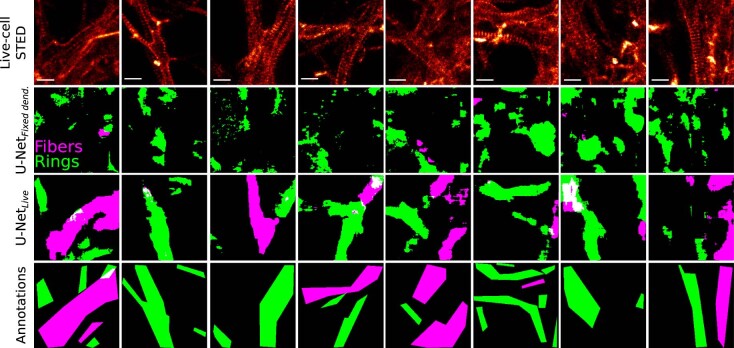
U-NetLive example results for the segmentation of F-actin nanostructures in live-cell STED images. Segmentation predictions by U-Net_*fixeddend*._^13^ and U-Net_*Live*_ on 8 representative images chosen from 28 annotated live-cell STED test images. Annotations were created for testing purposes and were not used for training U-Net_*Live*_. The U-Net_*Live*_ trained only on synthetic images from the *Translated F-actin dataset* succeeds in segmenting F-actin nanostructures on real STED images. Scale bars: 1 *μ*m.

The translated F-actin dataset, along with the expert annotations from the initial dendritic F-actin dataset, is used to train the U-Net_Live_ segmentation network to segment F-actin structures in images from the live-cell domain without requiring annotation of the live F-actin dataset (Fig. 3d and Supplementary Fig. 11). To confirm that training on synthetic domain-adapted images generalizes to real live-cell STED images, the area under the receiver operating characteristic (AUROC) was computed between the U-Net_Live_ segmentation masks and manual ground-truth annotations generated on 28 images by an expert in a user study (0.76 for rings and 0.83 for fibres; Extended Data Fig. 2 and Supplementary Figs. 12 and 13). In comparison, when applied to live-cell STED, the U-Net_fixed_ trained only on real images of fixed neurons achieves an AUROC of only 0.60 and 0.59 for the segmentation of rings and fibres, respectively. Thus, domain adaptation with TA-CycleGAN enables the use of synthetic images to train a modality-specific segmentation network (here U-Net_Live_) when no real annotated dataset is available for training. This facilitates the cumbersome step in the training of any supervised machine learning method: creating data-specific annotations. We next train TA-GAN_Live_ for resolution enhancement of live F-actin confocal images using the live F-actin dataset and the pretrained U-Net_Live_ as the auxiliary task network (Fig. 3e and Methods). The annotations generated by the U-Net_Live_ are used to compute the generation loss. Thus our image translation approach allows to train a TA-GAN to generate synthetic images from live-cell confocal images of F-actin in neurons (TA-GAN_Live_) as well as a segmentation network adapted to the live-cell imaging domain (U-Net_Live_), without the need to annotate the live F-actin dataset (Fig. 3f and Supplementary Fig. 14).

#### Automated modality selection with TA-GAN

**Fig. 4:**
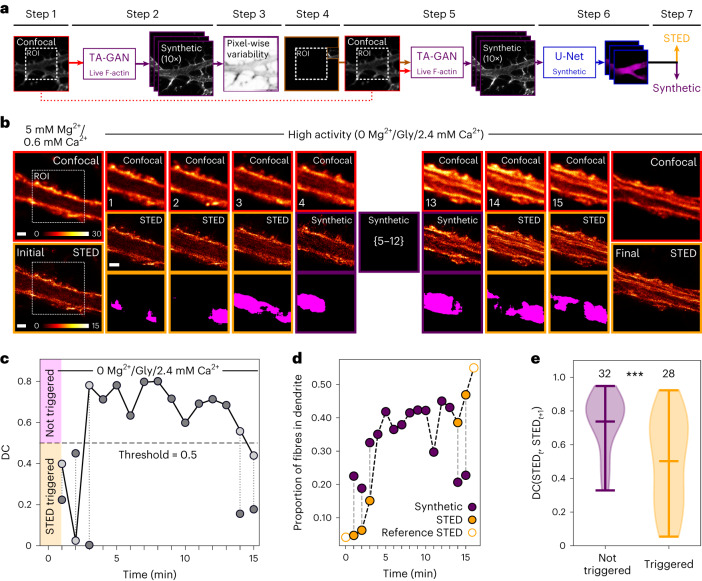
Monitoring change with the TA-GAN_Live_. **a**, Step-by-step imaging-assistance pipeline using the TA-GAN_Live_ in the live-cell acquisition loop. **b**, Live-cell imaging of dendritic F-actin before (initial), during (frames 1–15) and after (final) application of a stimulation solution (0 Mg^2+^/Gly/2.4 mM Ca^2+^). Shown are the confocal (red, top row), synthetic (purple, middle row) and real (orange, middle row) STED images when acquired, and corresponding segmentation masks for F-actin fibres (magenta, bottom row). The series was chosen as a representative example from a total of 72 series. Colour bars: raw photon counts. **c**, The DC at each time point measured between the current synthetic image and the last acquired reference STED image for the sequence shown in **b**. Dark grey points indicate that the last acquired real STED (used as reference) is from a previous time step and light grey points connected with a vertical dashed line indicate that a new STED is acquired at this time step, and the DC is recomputed with this new reference. **d**, Proportion of dendritic F-actin fibres at each time point segmented by the U-Net_Live_ on either the real STED (orange) or the synthetic STED (purple) images. When a real STED acquisition is triggered, the proportion of fibres in both images is compared (dotted line). Initial and final reference STED images (empty orange circles) are acquired at each round. **e**, The DC is computed for the F-actin fibre segmentation on control sequences of two consecutive real STED images (time points *t* and *t* + 1)). The segmentation of the STED_*t*_ image is used as reference and the DC is computed with the segmentation mask on the STED_*t*+1_ image. When a real STED image acquisition would not have been triggered by the threshold-based approach, the DC between the segmentation masks of the two real STED is higher. *n* = 60 control sequences of two consecutive confocal–STED pairs. Violin plots show the minimum, maximum and mean. Statistical analysis: two-sided Mann–Whitney *U* test^62^ for the null hypothesis that the two distributions are the same (***P = 0.0004). Scale bars, 1 μm. Source data

**Fig. 5:**
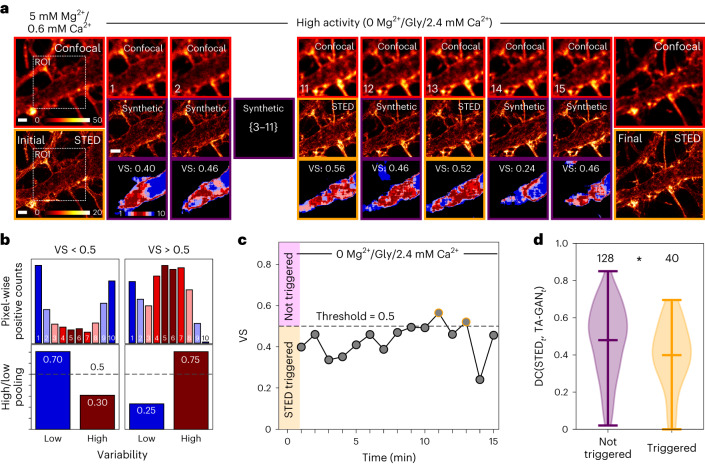
Monitoring prediction variability with the TA-GAN_Live_. **a**, Live-cell imaging of dendritic F-actin using the same stimulation as in Fig. 4. The TA-GAN_Live_ variability maps are shown on the bottom row. The series was chosen as a representative example from a total of 87 series. Colour bars: raw photon counts. **b**, Example histograms of the pixel-level positive counts over the segmentation of ten synthetic images (top) and high- and low-variability pooling. On the left, the VS is below 0.5 (dashed line, no trigger); on the right, the VS is above 0.5 (STED triggered). **c**, The VS at each time point for the sequence shown in **a**. When the VS is above 0.5, the number of high-variability pixels exceeds the number of low-variability pixels (**b**, VS > 0.5, right), which triggers the acquisition of a real STED image (orange circles). **d**, The DC is computed between the segmentation masks of synthetic and real STED image from the same time point (*n* = 168 pairs of real and synthetic images). When an STED acquisition would have been triggered using the VS criterion, the DC between the two corresponding images is lower. Violin plots show the minimum, maximum and mean. Statistical analysis: two-sided Mann–Whitney *U* test for the null hypothesis that the two distributions are the same (*P = 0.014). Scale bars, 1 μm. Source data

**Extended Data Fig. 3:**
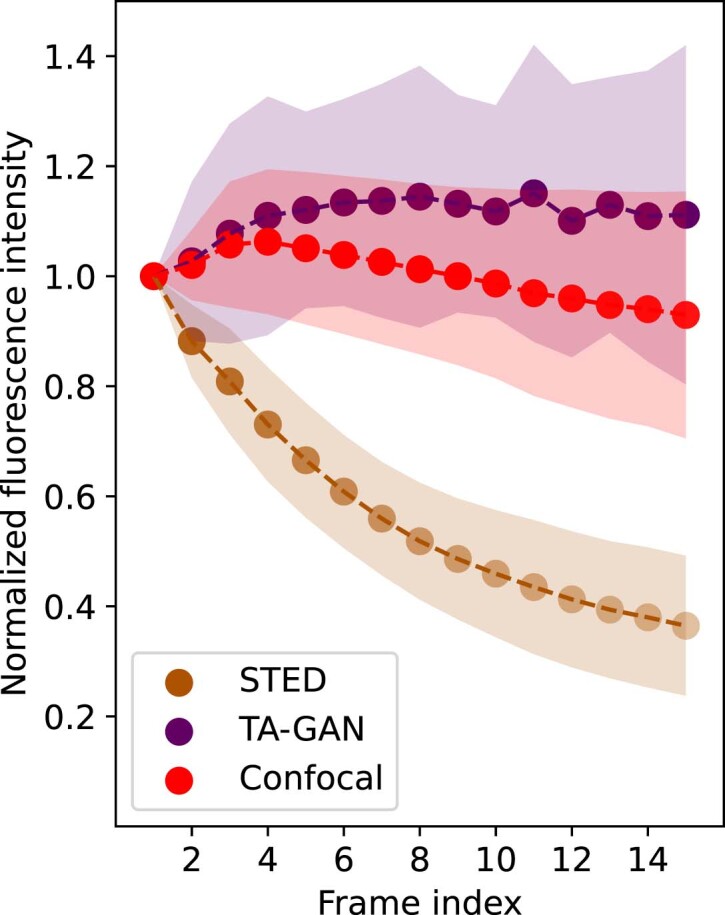
Comparison of photobleaching effects for consecutive confocal and STED acquisitions. Normalized fluorescence intensity after 15 confocal acquisitions (red, N=45 regions) and, associated synthetic STED signal (purple, N=45 regions) over the central ROI (300 × 300 pixels) in comparison to acquisitions using the STED modality at each frame (orange, N=45 regions). Dots show the average and shaded regions cover the standard deviation. The TA-GAN_*Live*_ predictions compensate for the fluorescence intensity decrease in the synthetic STED images. The 15th consecutive STED image has 36 ± 12 % of the initial STED image intensity and 92 ± 16 % for the sequence of confocal images for the corresponding TA-GAN generated images. Source data

Optimizing light exposure is of particular concern for live-cell imaging, where multiple acquisitions over an extended period of time might be required to observe a dynamic process. In super-resolution microscopy, repeated imaging with high-intensity illumination can cause photobleaching, which quickly diminishes the signal quality (Supplementary Fig. 15 and Extended Data Fig. 3). We evaluate how the integration of the TA-GAN_Live_ in the acquisition loop of an STED microscope can guide imaging sequences for time-lapse live-cell microscopy. We apply our approach to detect the activity-dependent remodelling of dendritic F-actin from periodical rings into fibres in living neurons, which was previously observed in fixed neurons but could not be monitored in living neurons due to technical limitations^13^. For a given image acquisition sequence, we first acquire a confocal image (Fig. 4a, step 1). We next use a Monte Carlo dropout approach^47^ to generate ten possible synthetic STED images with the TA-GAN_Live_. We apply a different random dropout mask for each image generated (Fig. 4a, step 2). This use of MC dropout with GANs has been previously demonstrated on natural images^48,49^ and serves as an estimation of the variability of TA-GAN_Live_ over the generated nanostructures. We next measure the optical flow between the ten synthetic images (Fig. 4a, step 3, and Methods). The subregion with the highest mean optical flow is acquired with the STED modality (Fig. 4a, step 4) and given as an input to the TA-GAN_Live_ together with the corresponding confocal image of the full field of view (FOV; Fig. 4a, step 5). This step helps to minimize the effect of signal variations encountered in live-cell imaging. The TA-GAN_Live_ generates, with different dropout masks, ten new synthetic images of the region of interest (ROI), which are segmented by the U-Net_Live_ to detect the presence of F-actin fibres (Fig. 4a, step 6). The segmentation predictions of the U-Net_Live_ for the synthetic images are used to decide whether or not a real STED image should be acquired at a given time point (Fig. 4a, step 7). The acquisition of a complete frame using the STED modality is triggered when either (1) the segmentation prediction on the synthetic STED image is different from the one obtained on the last acquired real STED image (Fig. 4b–e) or (2) there is high variability in the segmentation predictions on the ten synthetic STED images (Fig. 5 and Methods).

**Extended Data Fig. 4:**
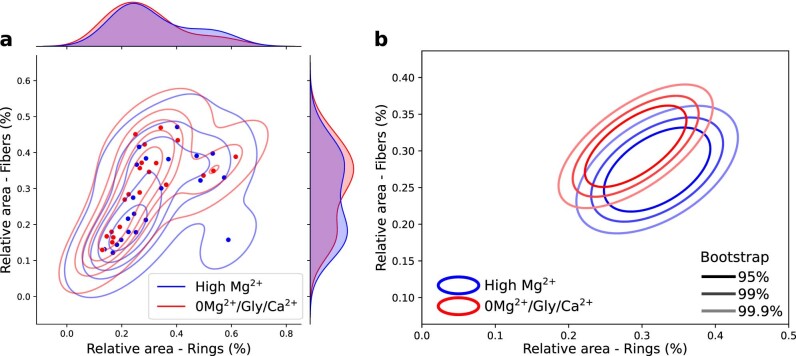
Observation of F-actin remodeling in living cells. **a**, Kernel density estimate of the F-actin fibres and rings dendritic area distribution for after 30 minutes in a solution reducing neuronal activity (high Mg^2+^/low Ca^2+^, blue) or following a stimulation (0Mg^2+^/Glu/Ca^2+^, from *t* = 1-15min, red). **b**, Bootstrapped distributions of the results shown in **a**,. Shown are the regions comprising 95%, 99% and 99.9% of the data point distribution. Following the 0Mg^2+^/Glu/Ca^2+^ stimulation, we observe a small increase in the proportion of F-actin fibres and a decrease in the proportion of rings. High Mg^2+^ N=21, 0Mg^2+^/Glu/Ca^2+^ N=21. Source data

For the first acquisition scheme we calculate at each time point the mean DC between the segmentation masks from the ten generated synthetic images and the last real STED image (Fig. 4b and Supplementary Figs. 16 and 17). A new STED image is acquired if the mean DC between the synthetic and the reference real STED images is below a predefined threshold of 0.5 (Fig. 4c). Using paired confocal and real STED images acquired at the end of the imaging sequence (15 min), we measure an increase in the proportion of F-actin fibres in living neurons (Fig. 4b, last frame, and Extended Data Fig. 4). On the basis of control sequences of two consecutive STED and confocal images pairs, we measure that the segmentation masks of those real STED images are more similar for sequences that would not have triggered a new real STED image acquisition, indicating that STED acquisitions are triggered at time points of higher biological change (Fig. 4e and Supplementary Fig. 18a,b). The value of the DC threshold is chosen based on preliminary imaging trials and previous knowledge about the remodelling extent and dynamics, which, depending on the experimental context, is not always available before the imaging experiment. With this acquisition scheme an average of 1.6 STED images are acquired per sequence (15 confocal images per sequence, 72 sequences). It reduces the light dose in average by 89% in the central ROI compared with acquiring 15 consecutive STED images.

We developed a second method to trigger STED image acquisitions, which is based on the variability in the predictions of the TA-GAN_Live_. This approach is particularly useful when not enough previous knowledge on the expected structural change is available to define a threshold for the DC before the experiment. With this acquisition scheme, for each confocal acquisition, we measure the pixel-wise variability of the segmentation predictions on the ten generated synthetic STED images (Fig. 5a). Pixels predicted to belong to the same class (fibres or not fibres) in ≥80% of the synthetic images are defined as low-variability pixels, and pixels predicted to belong to the same class in <80% by the TA-GAN_Live_ are defined as high-variability pixels (Fig. 5b). The proportion of high-variability pixels corresponds to the variability score (VS; Supplementary Fig. 19). When the VS is higher than 0.5 for the ROI, a full STED image is acquired (Fig. 5 and Supplementary Fig. 20). We validate the VS criterion on a set of real STED reference images and their corresponding synthetic counterparts. On these images, we measure a higher DC between the segmentation masks when a real STED image acquisition would not have been triggered by the VS threshold (Fig. 5d and Supplementary Fig. 18c,d). This indicates that the VS is a good indicator of the similarity between the real and synthetic STED image at a given time point. This approach can be beneficial to detect unexpected patterns and rare events. An average of 3.8 STED images were acquired for each sequence (87 sequences) using the variability-based triggers, which reduces the light dose in average by 74% in the central ROI compared with acquiring an STED image of the ROI at every frame. For both approaches, modulation of the STED modality acquisition frequency can be achieved by adapting the DC or VS thresholds. The resulting frame rate with TA-GAN assistance is comparable to acquiring sequences of paired confocal and STED images (Extended Data Table 1).

### Discussion

**Extended Data Fig. 5:**
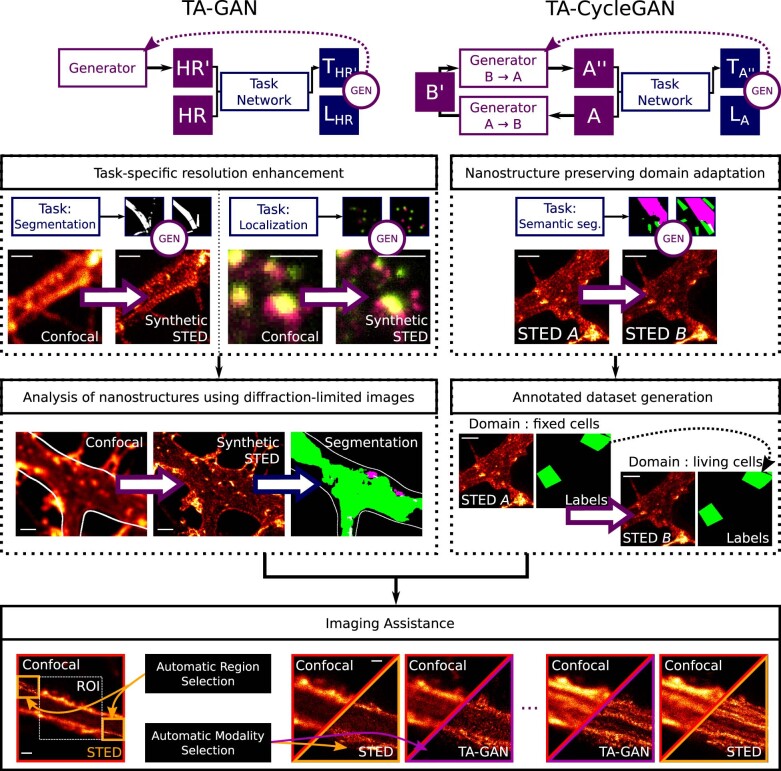
Graphical abstract. The proposed model has two general use cases: TA-GAN, for paired datasets, and TA-CycleGAN, for unpaired datasets. *Top-left:* The TA-GAN uses a task adapted to each dataset for accurate resolution enhancement. The generation loss (GEN circle) is computed from the comparison between the output of the task network for the synthetic high-resolution image (${T}_{H{R}^{{\prime} }}$) and the labels obtained from the ground truth image (*L*_*HR*_). The loss is backpropagated to the generator (dashed arrow). *Middle-left:* The generated synthetic STED images are used to analyze the distribution of nanostructures that were not resolved in the original confocal image. *Top-right:* Domain adaptation using the TA-CycleGAN enables the generation of large annotated synthetic image datasets from a new domain, even if labels are only available in one domain. The generation loss (GEN circle) is computed from the comparison between the output of the task network for the image and the synthetic version (${T}_{{A}^{{\prime}{\prime} }}$) and the labels obtained from the input domain A image (*L*_*A*_). The loss is backpropagated to the generator (dashed arrow).*Middle-right:* Labeled datasets from domain A (e.g fixed cells) are adapted to the unlabeled domain B (e.g live cells) to obtain a labeled dataset from domain B, which can be used to train a super-resolution TA-GAN. *Bottom:* Both models can be used for microscopy acquisition guidance. The TA-GAN model, trained using a TA-CycleGAN generated dataset, can automatically identify regions and frames of interest from the low-resolution images. Automatic switching between low- and high-resolution imaging modalities is guided by the TA-GAN_*Live*_ predictions. Scale bars: 1 *μ*m.

We introduce TA-GAN for resolution enhancement and domain adaptation. We demonstrate its applicability to optical nanoscopy (Extended Data Fig. 5) and show that an auxiliary task assisting the training of a generative network improves the reconstruction accuracy of nanoscopic structures. The applicability of our method is demonstrated for paired confocal and STED microscopy datasets of F-actin in axons and dendrites, synaptic protein clusters, simulated nanodomains as well as for paired bright-field and SIM images of dividing *S. aureus* bacterial cells. We show that the TA-GAN method is flexible and can be trained with different auxiliary tasks such as binary segmentation, semantic segmentation and localization. For unpaired datasets, we introduce the TA-CycleGAN model and demonstrate how the structure-preserving domain adaptation opens up the possibility to create paired datasets of annotated images that cannot be acquired simultaneously. The synthetic STED images from the live-cell domain can be used to train a neural network that performs well for the segmentation of F-actin nanostructures in real STED images, without the need for manual re-annotations of the new live-cell imaging dataset. The TA-GAN for resolution enhancement in living neurons can be integrated into the acquisition loop of an STED microscope (Figs. 4 and 5). We validate how this TA-GAN model can be helpful in assisting microscopists by automatically taking decisions that optimize the photon budget and reduce photobleaching (Extended Data Fig. 3) in live-cell optical nanoscopy acquisition sequences. The TA-GAN increases the informative value of each confocal acquisition and automatically triggers the acquisition of an STED image only in the regions and time steps where this acquisition is informative due to variations (Fig. 4) or uncertainties in the predicted nanostructures (Fig. 5).

Future work in calibrating the network’s probabilistic output could lead to an improved quantification of its confidence. Multiple successive frames could also be given as input to the generator to introduce temporal information instead of using static frames individually. This could enable the generator to decode the rate of biological change and introduce this knowledge to the next frame prediction, leading to smoother transitions between synthetic images. The TA-GAN model, as presented here, enables the visualization of biological dynamics over longer sequences with reduced photobleaching effects. Thus, TA-GAN-assisted STED nanoscopy can guide microscopists for optimized acquisition schemes and reduced light exposure.

## Methods

### Sample preparation and STED microscopy

#### Cell culture

Dissociated Sprague Dawley rat hippocampal neurons were prepared as described previously^13,50^ in accordance with and approved by the animal care committee of Université Laval. For live-cell STED imaging, the dissociated cells were plated on poly-d-lysine–laminin-coated glass coverslips (18 mm) at a density of 322 cells per mm^2^ and used at 12–16 days in vitro.

#### STED microscopy

Live-cell super-resolution imaging was performed on a four-colour STED microscope (Abberior Instruments) using a 40 MHz pulsed 640 nm excitation laser, an ET685/70 (Chroma) fluorescence filter and a 775 nm pulsed (40 MHz) depletion laser. Scanning was conducted using a pixel dwell time of 5 μs, a pixel size of 20 nm and an 8 line repetition sequence. The STED microscope was equipped with a motorized stage and auto-focus unit. The imaging parameters used are described in Supplementary Table 1.

The cultured neurons were pre-incubated in HEPES buffered artificial cerebrospinal fluid (aCSF) at 33 °C with SiR-actin (0.5 μM, SpiroChrome) for 8 min and washed once gently in SiR-actin-free media. Imaging was performed in HEPES buffered aCSF of 5 mM Mg^2+^/0.6 mM Ca^2+^ (NaCl 98 mM, KCl 5 mM, HEPES 10 mM, CaCl_2_ 0.6 mM, glucose 10 mM, MgCl_2_ 5 mM) using a gravity-driven perfusion system. Neuronal stimulation was performed with an HEPES buffered aCSF containing 2.4 mM Ca^2+^, glycine and without Mg^2+^ (NaCl 98 mM, KCl 5 mM, HEPES 10 mM, glycine 0.2 mM, CaCl_2_ 2.4 mM, glucose 10 mM). Solutions were adjusted to an osmolality of 240 mOsm per kg and a pH of 7.3.

### Datasets

#### Axonal F-actin dataset

The publicly available axonal F-actin dataset^13^ was used to train the TA-GAN_Ax_ for confocal-to-STED resolution enhancement of axonal F-actin nanostructures using the binary segmentation of F-actin rings as the auxiliary task. The original dataset consisted of 516 paired confocal and STED images (224 × 224 pixels, 20 nm pixel size) of axonal F-actin in fixed cultured hippocampal neurons from ref. ^13^. Thirty-one images from the original dataset were discarded for not containing annotated axonal F-actin rings. The remaining images were randomly split into a training set (377 images), a validation set (56 images) and a testing set (52 images), which was not used for training. The manual polygonal bounding box annotations of the axonal F-actin periodical lattice (F-actin rings) from the original dataset were retained (Fig. 1b).

#### Dendritic F-actin dataset

The publicly available dendritic F-actin dataset was used to train the TA-GAN_Dend_ for confocal-to-STED resolution enhancement of dendritic F-actin nanostructures using the semantic segmentation of F-actin rings and fibres as the auxiliary task. The dendritic F-actin dataset was also used to train the TA-CycleGAN for domain adaptation. The original dataset from ref. ^13^ was split into a training set (304 images), a validation set (54 images) and a testing set (26 images, 12 for low activity and 14 for high activity). We used the same testing split as the original publication to compare the segmentation results over the same images (Supplementary Fig. 10). The dataset consists of paired confocal and STED images of the dendritic F-actin cytoskeleton in fixed cultured hippocampal neurons, which had been manually annotated using polygonal bounding boxes. The training and validation crops were taken from large STED images (between 500 × 500 pixels and 3,000 × 3,000 pixels, 20 nm pixel size) using a sliding window of size 224 × 224 pixels with no overlap. If less than 1% of the pixels of the crop were annotated as containing a structure of interest (F-actin rings and/or fibres), the crop was discarded from the set. This operation resulted in 4,331 crops for training and 659 crops for validation.

#### Simulated nanodomains dataset

We used the pySTED image simulation platform^39^ to create a simulated dataset of nanodomains within a dendritic spine. The pySTED simulator requires as input a matrix providing the position and number of fluorescent molecules for each pixel in the FOV, referred to as a datamap. Each datamap (64 × 64 pixels or 1.28 × 1.28 μm) consisted of a mushroom spine-like shape (between 0.12 μm^2^ and 0.48 μm^2^) containing *N* (1–6) regions (20 × 20 nm) with a higher fluorophore concentration, which we refer to as nanodomains. In the majority of the images, the simulated STED modality was required to resolve all nanodomains. The position of the nanodomains was randomly distributed on the edge of the synapse (<140 nm away from the edge) with a minimal distance of 40 nm between nanodomains. We allowed random rotation and translation of the spine making sure that the nanodomains were kept within the FOV. For training, we generated a total of 1,200 simulated datamaps (200 for each number of nanodomains). The training and validation datasets were split using a 90/10 ratio. The localization maps are matrices of size 64 × 64 pixels, where the value of each pixel is the cubic root of the distance to the closest nanodomain. Two testing datasets were created. The first consisted of 75 simulated datamaps with different numbers of nanodomains (2–6, 15 images per number of nanodomains). The second consisted of 80 images with two nanodomains, where the distance between the pair of nanodomains varies from 40 nm to 450 nm.

#### Synaptic protein dataset

The publicly available synaptic protein dataset consists of paired two-colour STED and confocal images of the synaptic protein pair PSD95 (postsynapse) and bassoon (presynapse) in fixed hippocampal neurons obtained from ref. ^41^. The dataset was split into a training set (32 images), a validation set (2 images) and a testing set (9 images). The confocal and STED images from the training and validation sets were first registered using the pipeline presented in Supplementary Fig. 21, resulting in 690 crops for training and 35 crops for validation. The segmentation maps were generated by automatically segmenting the STED images using wavelet transform decomposition^42^ with the same parameters (scales 3 and 4) as in ref. ^41^. No segmented clusters were discarded based on size or position, following the intuition that even the smallest structures should be generated. The localization maps were created from a black image by placing a white pixel at the position of the intensity-weighted centroid of each segmented cluster, and then applying a Gaussian filter with a standard deviation of 2 (Supplementary Fig. 22).

#### *S. aureus* dataset

We used the bright-field images and the corresponding SIM images from the publicly available *S. aureus* dataset for segmentation from ref. ^44^. This dataset includes 12 images (6 for training, 1 for validation and 5 for testing) with manual whole-cell annotations. The bright-field images (80 nm per pixel) were rescaled to the size of the SIM images (40 nm per pixel) using bilinear interpolation, and the cell annotations were rescaled using nearest-neighbour interpolation. The whole-cell annotations were converted to binary segmentation maps with pixel values of 0 for background and 1 for cells. These whole-cell segmentation maps were used as LR annotations. HR annotations highlighting the cell division boundary were generated from the SIM images. To generate the HR annotations, we first applied a Sobel filter to the SIM images to find the outer and inner edges of the cells, followed by a Gaussian filter with a standard deviation of 1. We next applied a threshold corresponding to 20% of the maximum value of the filtered result. This resulted in a binary mask of the boundary between dividing cells as well as of the cell outer membrane. We similarly applied a Sobel filter to the LR annotations, followed by a Gaussian filter with a standard deviation of 1 and applied a threshold of 0 to generate a binarized cell border mask. The binarized cell border mask was subtracted from the mask of the outer and inner cell borders to generate the final HR annotations.

The training crops were generated using a sliding window of size 256 × 256 pixels with an overlap of 128 pixels. Crops were discarded if they contained less than 3% annotated pixels. The validation crops were generated using the same sliding window method, but for a size of 128 × 128 pixels without overlap. All validation crops were considered, regardless of the percentage of annotated pixels. The resulting dataset comprised 202 training crops and 64 validation crops.

#### Live F-actin dataset

The live F-actin dataset was acquired for this study and was used to train: (1) the TA-CycleGAN for live and fixed domain adaptation and (2) the TA-GAN_Live_. The live F-actin dataset consists of 800 paired STED and confocal images of F-actin stained with the fluorogenic dye SiR-actin (Spirochrome) in living hippocampal cultured neurons (Supplementary Table 1). The dataset was split into a training set (753 images) and a validation set (47 images). The images were of variable size (from a minimum width of 2.76 to a maximum of 49.1 μm, pixel size is always 20 nm).

#### Translated F-actin dataset

The translated F-actin dataset was used to train the TA-GAN_Live_. This dataset corresponds to the dendritic F-actin dataset adapted to the live-cell STED imaging domain using the TA-CycleGAN for fixed-to-live domain adaptation. It contains the same number of images, the same training, validation and testing splits, and the same image characteristics (crop size, pixel size, annotations) as the dendritic F-actin dataset.

### TA-GAN training procedure

The TA-GAN was developed from the cGAN model for image-to-image translation pix2pix^35^, available at https://github.com/junyanz/pytorch-CycleGAN-and-pix2pix. All the functions for training and testing TA-GAN use pytorch^51^ (1.0.0), torchvsion (0.2.1), numpy (1.19.2), Pillow (8.3.1), tifffile (2020.0.3), scipy (1.5.4) and scikit-image (0.17.2). Comparable methods using cGANs for enhancing the resolution of microscopy images are trained using pixel-wise generation losses to compare the generated image with the ground truth, such as MSE^5^, absolute error^15,16^ or structural similarity index^17,18^. For the TA-GAN, the generation loss is computed by comparing the output of an auxiliary task network applied on the real (ground truth) and generated (synthetic) images (Fig. 1a). The other standard losses for conditional GANs^35^ are also used for TA-GAN: the discrimination losses for the classification of the real and generated images, and the GAN loss for the misclassification of generated images. The networks (generator, discriminator and task network) are optimized using the Adam optimizer with momentum parameters *β*_1_ = 0.5 and *β*_2_ = 0.999 for all TA-GAN models. We follow the same approach as the pix2pix paper^35^: at each epoch we alternate between one gradient descent step on the discriminator, then one step on the generator, then one step on the task network. Supplementary Table 2 summarizes the settings for the resolution-enhancement experiments presented in this paper, and Supplementary Table 3 presents the hyperparameters used for training the TA-GAN for each of these experiments.

#### TA-GAN training with segmentation auxiliary tasks

The TA-GAN_Ax_, TA-GAN_Dend_, TA-GAN_Syn_ and TA-GAN_SA_ were trained for resolution enhancement using the segmentation of subdiffraction biological structures as the auxiliary task. The output of the segmentation network was compared with the ground-truth annotations using an MSE loss. The loss computed from the real STED image (task loss, TL in Fig. 1a) was backpropagated to the segmentation network to optimize its weights, and the loss from the synthetic STED image (GEN) optimizes the generator. The other losses computed were standard cGAN losses: the GAN loss (GAN, misclassification of synthetic images as real images), the discriminator losses (DR, classification of real images as real, and DG, classification of generated images as synthetic). The validation losses were not used for early-stopping because of the adversarial nature of GANs. The validation images were instead used as a qualitative assessment of the training progress to select the best iteration for testing the model.

For the TA-GAN_Ax_, the auxiliary task was the segmentation of axonal F-actin rings. The output of the auxiliary task network was the predicted segmentation maps of F-actin rings. The spatial resolution of the real and synthetic images were not significantly different (Supplementary Table 4 and Supplementary Fig. 23).

For the TA-GAN_Dend_, the auxiliary task was the semantic segmentation of dendritic F-actin rings and fibres. The output of the auxiliary task network was a two-channel image, with the predicted segmentation maps of F-actin rings in the first channel and of F-actin fibres in the second channel. The spatial resolution of the real and synthetic images were not significantly different (Supplementary Table 4 and Supplementary Fig. 23).

For the TA-GAN_SA_, the auxiliary task was either whole-cell segmentation (LR annotations) or the segmentation of the boundary between dividing cells (HR annotations). The output of the auxiliary task network is a one-channel image with the predicted segmentation maps of either whole cells or the dividing cell boundaries, respectively.

For the TA-GAN_Syn_ trained using a segmentation task, the output of the segmentation network is a two-channel image with the predicted segmentation maps of PSD95 clusters in the first channel and bassoon in the second channel. The spatial resolution of the real and synthetic images were not significantly different (Supplementary Table 4 and Supplementary Fig. 23).

#### TA-GAN training with localization auxiliary tasks

The TA-GAN_Syn_ and TA-GAN_Nano_ for confocal-to-STED resolution enhancement were trained using a localization network to compute the generation loss. The localization network took an STED image as input to output a map of dots indicating the intensity-weighted centroids of all detected clusters in the STED image.

The TA-GAN_Syn_ was trained on the synaptic protein dataset using the two-channel confocal image rescaled and registered to the STED image. The generation loss (GEN in Fig. 1) was the MSE between the weighted centroids of the real STED image and the localization predictions from the task network on the synthetic image. The spatial resolution of the real and synthetic images were not significantly different (Supplementary Table 4 and Supplementary Fig. 23).

TA-GAN_Nano_ was trained on the simulated nanodomain dataset using the simulated confocal image as input. The generation loss was the MSE between the localization maps from the ground-truth datamaps and the localization predictions from the task network on the synthetic image.

#### TA-CycleGAN training for domain adaptation

The TA-CycleGAN model was developed from the CycleGAN model^35^. As for the standard CycleGAN, the TA-CycleGAN consists of four networks: two generators (one that translates the domain of fixed-cell STED imaging (F) into the domain of live-cell STED imaging (L), and one that translates domain L into domain F), and two discriminators (one for domain F, the other for domain L), which are combined with a fifth network, the task network (Fig. 3c). The TA-CycleGAN was applied to non-paired images, where the prediction of the generator for a given input cannot be compared with a corresponding ground truth. Instead, the generated synthetic image was passed through a second generator and converted back to the input domain where it was compared with the initial image (ground truth) for the computation of losses.

The TA-CycleGAN for fixed-to-live domain adaptation was trained using two datasets: the dendritic F-actin dataset (F) and the live F-actin dataset (L). The auxiliary task was the semantic segmentation or F-actin rings and fibres on the dendritic F-actin dataset, for which manual bounding box annotations were available^13^. The U-Net_fixed-dend_ was already optimized for the semantic segmentation of F-actin rings and fibres in fixed-cell STED images^13^. The generation loss was the MSE between the U-Net_fixed-dend_ segmentation prediction on the real fixed-cell image (fixed) and the end-of-cycle fixed-cell image (fixed_rec_) (Fig. 3c).

### Training procedures of resolution enhancement and denoising baselines

#### Enhanced super-resolution generative adversarial network

ESRGAN x4 (ref. ^33^) is a state-of-the-art method for upsampling natural images. ESRGAN was implemented from the public GitHub repository (https://github.com/xinntao/Real-ESRGAN). We fine-tuned ESRGAN on two of our datasets, the axonal F-actin dataset and the simulated nanodomains dataset, using the code and pretrained weights released with the most recent iteration of the model, Real-ESRGAN^34^. For both datasets, the input of the model is the confocal image, and the target output is the corresponding STED image upsampled four times using nearest-neighbour interpolation. Even though the confocal and STED images are the same size, the upsampling had to be kept in the model to use the pretrained weights. ESRGAN was fine-tuned for 50,000 iterations. The model was applied to the validation images to ensure training had converged after 50,000 iterations. All default parameters proposed by the authors were used, except for the input crop size and batch size (128 pixels and 4 for the axonal F-actin dataset, 64 pixels and 16 for the simulated nanodomains dataset).

#### Content-aware image restoration

CARE^16^ uses a U-Net for deblurring, denoising and enhancing fluorescence microscopy images. CARE was implemented from the public GitHub repository (https://github.com/CSBDeep/CSBDeep). We used the standard CARE network for image restoration and enhancement. The residual U-Net generator was optimized from scratch on our datasets. The original CARE model does not use data augmentation, as it is trained on unlimited simulated images. We augmented our datasets before training the CARE models so that the number of training images is similar to the one used for the original model trained on simulated images (8,000 synthetic pairs of 128 × 128 pixels). For the axonal F-actin dataset, each image from the training set is augmented 32 times by cropping the four corners into 128 × 128 crops and applying the 8 possible flips and rotations to each corner crops. The 377 224 × 224 images were augmented into 12,064 different crops. For the simulated nanodomains dataset, the 64 × 64 images were too small to be further cropped, but were instead augmented 8 times using flips and rotations. The 1,080 training images were augmented into 8,640 different images. The patience parameter for the learning-rate decay function was adjusted from 10 to 20 epochs after noticing that the learning rate was reduced too abruptly to allow the training loss to properly converge. Except for the patience of the learning-rate decay function, default hyperparameters were used and the model was trained for 100 epochs using a mean absolute error loss. The epoch that reached the lowest validation loss was used for testing.

#### Residual channel attention network

RCAN^52^ uses residual channel attention networks to increase the resolution of natural images. 3D-RCAN^15^ adapts the original model to denoise and sharpen fluorescence microscopy image volumes. We used the code implemented with TensorFlow and Keras from the publicly available GitHub repository (https://github.com/AiviaCommunity/3D-RCAN). We used the same patch size as for training the TA-GAN_Ax_ (128 × 128 pixels) and the TA-GAN_Nano_ (64 × 64 pixels). We trained different RCAN models using configurations of hyperparameters that were inspired by both the two-dimensional (2D)^52^ and the three-dimensional (3D)^15^ versions. We first trained a model on the axonal F-actin dataset with the hyperparameters from the 2D RCAN version. Even though both training and validation losses had converged, the output obtained with the weights from the epoch of lowest validation loss (epoch 205 out of 1,000) is an unrecognizable and smoothed version of the input. We hypothesize that this version of the model is too deep (15 million trainable parameters) for the number of training images. We trained a second version of RCAN using the hyperparameters from ref. ^15^. The loss when training this model quickly converges to a minimum (epoch 34 out of 300) and the resulting images are smoothed versions of the input confocal image. This simplified version of RCAN might be too lightened for the 2D context. The architecture that ended up performing the best with our datasets mixes hyperparameters from both implementations. (1) We used 2D convolutions because our images are 2D, as in RCAN. (2) We set the number of residual groups to 10 in the residual in residual structure, as in 3D-RCAN. (3) The residual channel attention blocks were set to 20, as in RCAN. (4) We set the number of convolution layers in the shallow feature extraction and residual in residual structure to 32, as in 3D-RCAN. (5) We set the reduction ratio to eight as in 3D-RCAN. (6) The upscaling module was removed because the confocal and STED images are the same size, as is the case for 3D-RCAN. This RCAN model was trained for 1,000 epochs for both datasets to ensure convergence of the validation loss. The model reaching the lowest validation loss (epoch 838 for the simulated nanodomains dataset, epoch 398 for the axonal F-actin dataset) was used for testing.

#### cGAN for image-to-image translation

pix2pix^35^ is a state-of-the-art method for image-to-image translation in natural images. It was implemented with Pytorch from the publicly available GitHub repository (https://github.com/junyanz/pytorch-CycleGAN-and-pix2pix). The TA-GAN and pix2pix share the same architecture with or without the task assistance. For each experiment, the same hyperparameters and datasets as for the TA-GAN were used for training (Supplementary Table 3), replacing only the generation loss with a pixel-wise MSE loss between the ground-truth and generated STED images. The results from this baseline are compared to the TA-GAN for all fixed-cell datasets.

#### Denoising convolutional neural network

The denoising convolutional neural network (DnCNN)^36^ is a state-of-the-art denoising method for natural images. The trained version of DnCNN^36^ available at https://github.com/yinhaoz/denoising-fluorescence was directly applied to our test images for all datasets (Supplementary Fig. 1). The datasets used in this study do not provide the required characteristics to retrain DnCNN (that is, lack of images with different noise levels); therefore, a published version of the DnCNN trained on the fluorescence microscopy denoising dataset^37^ was used as is. It was included as a baseline to show how the confocal-to-STED and bright-field-to-SIM transformations are not denoising tasks.

#### Noise2Noise

Noise2Noise^38^ is a state-of-the-art deep learning denoising method that does not require clean (denoised) data for training. Like DnCNN, we used the training version available at https://github.com/yinhaoz/denoising-fluorescence and directly applied it to the test images from our datasets, without retraining or fine-tuning (Supplementary Fig. 1).

### Evaluation of networks performance

#### Segmentation of F-actin nanostructures in synthetic STED images

The performance of the TA-GAN_Ax_ was measured on the images from the test set of the axonal F-actin dataset, which were held-out and not used for training the TA-GAN_Ax_ or the baselines. The MSE, PSNR and SSIM were computed between the ground-truth and synthetic STED images of the test set (Extended Data Fig. 1). In addition, U-Net_fixed-ax_, a U-Net that was trained to segment axonal F-actin rings on real STED images only^13^ (available at https://github.com/FLClab/STEDActinFCN), was used to produce segmentation masks of axonal F-actin rings on the real and synthetic STED image pairs (Extended Data Figs. 1 and 3), which were compared using the DC and IOU metrics. We used the trained weights provided and did not retrain U-Net_fixed-ax_ specifically for this work.

The performance of the TA-GAN_Dend_ was evaluated on the test set of the dendritic F-actin dataset, which was held-out and not used to train the TA-GAN_Dend_ or the U-Net_fixed-dend_. The U-Net_fixed-dend_, a U-Net that was trained for the semantic segmentation of dendritic F-actin rings and fibres on real STED images only^13^ (available at https://github.com/FLClab/STEDActinFCN), was used to segment the real and synthetic STED images. The segmentation masks of both F-actin rings and fibres were compared on the real and synthetic STED image pairs (Supplementary Fig. 10). We used the trained weights provided and did not retrain U-Net_fixed-dend_ specifically for this work.

#### Assessment of synaptic protein cluster morphology

The perimeter, eccentricity, area, distance to nearest neighbour from the same channel and distance to nearest neighbour from the other channel of the protein clusters from the synaptic protein dataset were measured in the confocal, STED images and synthetic images (Fig. 2 and Supplementary Fig. 7). The distribution of each morphological feature over all associated clusters from the test set images was computed using a Python library for Statistical Object Distance Analysis (pySODA)^41^ (Supplementary Fig. 7). A foreground mask was generated following ref. ^41^: applying a Gaussian blur (standard deviation of 10) on the sum of both STED channels, and thresholding the image using 50% of the mean intensity value. Only clusters from the foreground mask were considered for the analysis. The same parameters as in ref. ^41^, which were optimized for real STED images of synaptic protein clusters, were used for the analysis: wavelet segmentation scales of 3 and 4, a minimum cluster area of 5 pixels, and minimum cluster width and height of 3 pixels. The weighted centroids of the detected clusters were calculated on the raw STED images.

#### Classification of *S. aureus* cells

The TA-GAN_SA_ performance was evaluated using the classification of dividing bacterial cells, which is a task that cannot be achieved using only the bright-field images. A simple threshold optimization applied on bright-field images was not sufficient to classify the cells as dividing or not (Supplementary Fig. 9). A dividing bacterial cell is defined as having a clear boundary between the two dividing cells that can be identified in the SIM image. We trained the ResNet_SA_ using the SIM images (training set) and the HR annotations, to segment the dividing cell boundaries. The ResNet_SA_ is a ResNet-9 architecture trained for 200 epochs using an MSE loss, a learning rate of 0.0002 and the Adam optimizer. All real SIM images and synthetic SIM images generated from pix2pix_SA_, TA-GAN_SA_ trained with LR annotations and TA-GAN_SA_ trained with HR annotations are segmented by ResNet_SA_.

The dividing/non-dividing cells classification was based on the segmentation of the ResNet_SA_: (1) dividing if the segmentation mask contained at least 20 positive pixels and (2) non-dividing if the segmentation mask was empty for a given cell. For segmentation masks containing 1–19 pixels, the cells were identified as ambiguous and discarded. On the real SIM images test set, 251 cells were identified as non-dividing (single cells) and 159 as dividing (showing a clear boundary between the dividing cells).

#### User study for the segmentation of live F-actin images

A set of 28 STED images (224 × 224 pixels) from the live F-actin dataset test set was labelled by an expert using a Fiji^53^ macro to test the performance of the U-Net_Live_ trained on the domain-adapted dendritic F-actin dataset for the segementation of real live-cell STED images. In addition, a second set of 28 synthetic images, selected from the domain-adapted dendritic F-actin dataset was included in the user study. The expert was presented with an image from one of the two sets, without being informed whether the image was real or synthetic. For each image, the expert draws polygonal bounding boxes that enclosed all regions identified as F-actin rings and fibres.

#### User study for the localization of nanodomains

The positions of the nanodomains in the real and synthetic test images of the simulated nanodomains dataset were identified by an expert to compare the localization performance of the TA-GAN_Nano_ with the baseline methods. The expert was presented with an image without being informed whether the image was real or synthetic, or by which model it was generated. For each image, the expert selects the pixel identified as the centre of each nanodomain detected. To compute the F1 score, a detection is defined as a true positive if it is within 3 pixels of the ground-truth position of a nanodomain centre.

### TA-GAN-assisted live-cell STED microscopy

#### Training of the TA-GAN_Live_

The TA-GAN_Live_ for resolution enhancement of live-cell STED imaging was trained on the new and not previously annotated live F-actin dataset. The auxiliary task was the semantic segmentation of dendritic F-actin rings and fibres. The original live F-actin dataset did not include any manual annotations. To circumvent this limitation, the U-Net_Live_ segmentation network was pretrained on the domain-adapted dendritic F-actin dataset. The pretrained U-Net_Live_ was frozen during the TA-GAN_Live_ training and was used to compute the MSE generation loss between the segmentation prediction of the real and the synthetic STED images.

To better adapt to cell-to-cell signal variations and experimental variability in live-cell STED images, the input of the generator has three channels: (1) the confocal image, (2) a real STED subregion acquired in the vicinity of the ROI and (3) an image indicating the position of the STED subregion (Fig. 3e). Training using this three-channel input enables the generator to learn features from the STED subregion and turns the resolution-enhancement task into an image-completion task.

#### Training of the U-Net_Live_

The U-Net_Live_ was built around a U-Net-128 (ref. ^54^) architecture with batch normalization and two output channels (F-actin rings and fibres) for the segmentation of F-actin nanostructures in living neurons.

The training of the U-Net_Live_ required an annotated dataset of images of the live-cell domain. A random subset (2,069 training crops and 277 validation crops) of the dendritic F-actin dataset was translated into the live-cell domain using the generator_Live_ (Supplementary Fig. 11). This resulted in the domain-adapted F-actin dataset. The manual annotation from the fixed-cell images were associated with the corresponding synthetic images from the live-cell domain (Supplementary Fig. 11a).

Random crops of 128 × 128 pixels of the domain-adapted F-actin dataset and their corresponding annotations were used to train U-Net_Live_ on images of the live-cell domain. Horizontal and vertical flips were used for data augmentation. Due to class imbalance in the training set, the segmentation loss for fibres was weighted by a factor of 2.5, which reflected the ratio of total annotated pixels for each class. The U-Net_Live_ was trained for 1,000 epochs and the iteration with the lowest segmentation loss over the validation set was kept for further use and testing. The optimal threshold to binarize the segmentation prediction was determined as the value that reached the optimal DC over the validation set (−0.53 for the raw output predictions).

#### TA-GAN integration in the acquisition loop

The TA-GAN_Live_ trained for resolution enhancement for live-cell imaging was directly integrated in the imaging acquisition process of the STED microscope (Fig. 4a). At the beginning and at the end of each experiments, an FOV of 10 × 10 μm was selected and reference STED and confocal images were acquired. The reference images were used to monitor the dendritic F-actin activity-dependent remodelling in living neurons (Extended Data Fig. 4). Similarity measurements between the synthetic and real STED images do not show time-dependent changes in the generation accuracy over all imaging sequences (Supplementary Fig. 24).

For each time point, as a first step (Table 1), a confocal image of the ROI is acquired to serve as the input to the TA-GAN_Live_ for the generation of ten synthetic resolution-enhanced images of the ROI (step 2, Table 1). The ten synthetic images of steps 2 and 5 are generated using different random dropout masks created with the default dropout rate of 0.5 from ref. ^55^ and confirmed to be appropriate when applied on GANs by ref. ^49^.

**Table 1:** Steps performed at each time point for automated TA-GAN assistance

1	A confocal image of the FOV (10 × 10 μm) is acquired.
2	Using dropout, ten synthetic images of the FOV are generated.
3	A subregion (2 × 2 μm) of highest variability outside the ROI is identified with optical flow.
4	A real STED image of this subregion is acquired.
5	The confocal images of the FOV and of the STED subregion are used by the TA-GAN to produce ten new synthetic STED images of the FOV.
6	The fibres in the ROI (central region of 6 × 6 μm) are segmented by U-Net_Live_.
7	The segmentation predictions are used to decide if an STED image should be acquired based on either (1) the mean DC with the segmentation of the last acquired STED or (2) the variability between the ten segmentation predictions.

The third step is the selection of an STED subregion outside the ROI (step 3, Table 1), which is given as input, along with the confocal FOV, to the TA-GAN_Live_ to account for signal variation in live-cell imaging. In the fourth step, the STED subregion is acquired on the microscope. Finally, this subregion (step 5, Table 1) is given as input to the TA-GAN together with the confocal image as described in the previous section. The STED images generated by TA-GAN_Live_ more closely match the ground-truth STED ROI when an STED subregion is given as input along with the confocal FOV (Supplementary Fig. 25).

In our imaging-assistance framework, we choose for step 3 (Table 1) to compute the pair-wise optical flow (OF) between the ten synthetic images generated with the TA-GAN_Live_ using dropout. The OF is computed using a Python implementation of the Horn–Schunck method^56^ with the Python multiprocessing library, parallelizing the computations on eight central processing units to increase the computation speed and avoid delays. The OF is computed between each pair of the ten synthetic images (1–2, 2–3, …). To translate the pixel-wise OF to a region-wise maps, the 500 × 500 pixels OF image was downsampled to a 5 × 5 map using the mean of each 100 × 100 pixels region. The subregion with the highest mean displacement is imaged with the STED modality. We decided to use OF as a measure of disparity between the synthetic generations, but other measures (for example, standard deviation, SSIM, mean intensity) could be used for experiments where computation time needs to be minimized (Supplementary Table 5). The sequence of acquiring the full confocal (2.6 s), generating ten synthetic STED images (2.5 s), computing the OF (6.1 s), acquiring the STED subregion (1.3 s), generating ten synthetic STED images again (2.5 s) and taking the decision requires a total of around 15.0 s per 500 × 500 pixels regions (10 × 10 μm). In comparison, acquiring an STED image requires 13.6 s using the same parameters (pixel size, pixel dwell time and size of the FOV).

Steps 2, 3, 5 and 6 are computed with a graphics processing unit to avoid computation induced delays. To do so, the commands from steps 2, 3, 5 and 6 are sent from the microscope’s control computer to a graphics-processing-unit-equipped computer using the Flask^57^ web framework Python module, version 2.0.3. All automated acquisitions use the SpecPy Python library version 1.2.1 to interface with the Imspector software (Abberior Instruments).

### Live-cell imaging decision guidance using the TA-GAN

The TA-GAN_Live_ predictions are used for decision guidance on the optimal STED and confocal acquisition sequence and applied to the imaging of F-actin remodelling dynamics in cultured hippocampal neurons. For a region size of 6 × 6 μm (300 × 300 pixels), as used for the live-cell experiments, a confocal acquisition applies to the sample a photon dose of 1.168 × 10^13^ photons per second, compared with 1.543 × 10^18^ photons per second with STED. The TA-GAN assistance aims at reducing the light dose by limiting STED acquisitions to only the time points where a structural change is predicted.

#### TA-GAN assisted monitoring of expected structural change

The proof-of-concept experiment targets the expected activity-dependent remodelling of dendritic F-actin rings into fibres^13^. On the basis of previous findings, the area of F-actin fibres was expected to increase following a neuronal stimulation^13^. The structural remodelling is monitored by comparing the area of segmented F-actin fibres on the synthetic and the reference real STED images. F-actin fibres are segmented on the synthetic STED images by U-Net_Live_. At each time point, steps 1–5 are performed as described in Table 1. To decide, following step 5, whether or not an STED image of the full ROI should be acquired, ten synthetic images of the ROI (acquired with the confocal modality) are generated and segmented by the U-Net_Live_. The mean of the ten segmentation maps is compared with the segmentation map predicted for the last acquired real STED image (reference STED) using the DC metric. A low DC is indicative of changes in the F-actin nanostructures in respect to the reference STED. A full real STED image is acquired if the DC falls below a pre-established threshold of 0.5. The value of 0.5 was chosen by performing several trials on live-cell F-actin imaging. The value of the DC threshold should be adapted to the type of structural remodelling observed. Each time the acquisition of an STED on the full ROI is triggered, the STED reference image is updated for subsequent comparison of the segmentation maps.

#### Monitoring the TA-GAN_Live_ generator’s variability

The pixel-wise generator’s variability can also be monitored to trigger the imaging of a full ROI with the STED modality. At each time point, steps 1–5 are performed as described in Table 1. The ten synthetic images generated at step 5 are segmented by the U-Net_Live_, resulting in ten segmentation maps for F-actin rings and fibres. The ten segmentation maps of F-actin fibres are binarized and summed. Pixels in the summed segmentation prediction has a value between zero and ten (zero when the presence of fibres was predicted in none of the synthetic images and ten when it is predicted in all). The variability of the generator on the segmentation prediction is evaluated from the summed segmentation prediction. Low-variability pixels are the pixels having the same value for at least 80% of the predicted segmentation maps (values of 1–2 (no fibres), 9–10 (fibres), positive counts). High-variability pixels are those having positive counts in between three and eight, inclusive. The distribution of high- and low-variability pixels from the foreground (Fig. 4b) is compared for each image. Pixels with zero positive counts (mostly background) are not considered. The proportion of low-variability pixels in the foreground is defined as the variability score (VS). A VS below 0.5 corresponds to images for which the predictions of the U-Net_Live_ on the ten synthetic images are consistent for the majority of foreground pixels. If the VS is above 0.5, the ten synthetic STED images are not consistent and an STED acquisition is triggered. The threshold of 0.5 was chosen because it corresponds to the tipping point where the number of high-variability pixels exceeds the number of low-variability pixels.

### Reporting summary

Further information on research design is available in the Nature Portfolio Reporting Summary linked to this article.

### Supplementary information

Supplementary Information Supplementary Tables 1–5 and Figs. 1–25.

Reporting Summary

### Source data

Source Data Fig. 2b,e Morphological features measured on all clusters from the test set of the synaptic protein dataset (for confocal, STED, pix2pix, TA-GAN localization and TA-GAN segmentation); classification prediction for each individual cell of the test set of the *S. aureus* dataset (bright field, SIM, pix2pix, TA-GAN LR and TA-GAN HR).

Source Data Fig. 4c,d,e Dice coefficient computed for the 15 frames series shown in Fig. 4b; proportion of fibres measured in real and synthetic STED for the 15 frames series of Fig. 4b; dice coefficient computed from 60 pairs of consecutive STED control images.

Source Data Fig. 5c,d Number of high-VS pixels and low-VS pixels for the 15 frames series shown in Fig. 5a; VSs of 168 confocal/STED control pairs.

Source Data Extended Data Fig. 1 Metrics (MSE, SSIM, PSNR, IOU, DC) computed over the test images of the axonal F-actin dataset.

Source Data Extended Data Fig. 3 Normalized fluorescence measured over 15 consecutive acquisitions of STED, confocal and TA-GAN generated images for 45 control series.

Source Data Extended Data Fig. 4 Proportion of rings and fibres measured in the initial and final STED images for 21 series of TA-GAN assisted live-cell imaging.

## Figures and Tables

**Extended Data Table 1:**
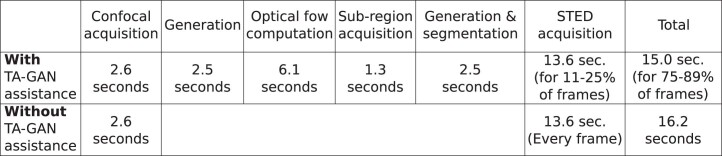
Processing time with and without the TA-GAN-assisted implementation

The number of STED images acquired with the TA-GAN assistance varies between sequences and averages 11% for the DC-based triggers and 25% for the variability-based triggers.

## Data Availability

The *S. aureus* dataset from refs. ^43,44^ is available at https://zenodo.org/record/5550933#.Y6IhFNLMJH4 (ref. ^43^) and https://zenodo.org/record/5551141#.Y6IjBdLMJH5 (ref. ^58^). The live F-actin dataset introduced here is available to download at https://zenodo.org/record/7908914 (ref. ^59^) and https://s3.valeria.science/flclab-tagan/index.html. Other datasets can be requested from their respective publications: the axonal F-actin dataset^13^, the dendritic F-actin dataset^13^, the synaptic protein dataset^41^ and the *S. aureus* dataset^43,44^. The processed versions of those datasets, as used to train the TA-GAN models, can be downloaded from https://s3.valeria.science/flclab-tagan/index.html. Sample test images are available at https://github.com/FLClab/TA-GAN in the ‘test’ subfolders of each dataset. Source data are provided with this paper.

## Code availability

The codes, trained weights and instructions on how to train, test and adapt for new experiments the TA-GAN model are available at https://github.com/FLClab/TA-GAN and 10.5281/zenodo.7908818 (ref. ^60^). The trained U-Net_Live_ model is available to download at 10.5281/zenodo.7909304 (ref. ^61^). The code, seeds and parameters used to generate the simulated nanodomains dataset are available at https://github.com/FLClab/TA-GAN and 10.5281/zenodo.7908818 (ref. ^60^).
