# Supplementary material for: Resolution enhancement with a task-assisted GAN to guide optical nanoscopy image analysis and acquisition
Source: Nat Mach Intell. 2023 Jul 27;5(8):830–44. doi: 10.1038/s42256-023-00689-3 (PMC10442226; doi:10.1038/s42256-023-00689-3)
Supplement: Supplementary file 1 — Supplementary Tables 1–5 and Figs. 1–25. [file 42256_2023_689_MOESM1_ESM.pdf]

nature machine intelligence

Article https://doi.org/10.1038/s42256-023-00689-3

Resolution enhancement with a task-assisted GAN to guide optical nanoscopy image analysis and acquisition

In the format provided by the authors and unedited

Supplementary Data

1 Supplementary tables **2**

Supplementary Table 1 - Imaging parameters 2
Supplementary Table 2 - Experimental settings 3
Supplementary Table 3 - Hyperparameters 3
Supplementary Table 4 - Resolution 4
Supplementary Table 5 - Generation performance and diversity 4

2 Supplementary figures **5**

Supplementary Figure 1 - Denoising baselines 6
Supplementary Figure 2 - TA-GAN$_{Ax.}$ results 7
Supplementary Figure 3 - TA-GAN$_{Ax.}$ autocorrelation curve 7
Supplementary Figure 4 - TA-GAN$_{Nano.}$ results (quantified) 9
Supplementary Figure 5 - TA-GAN$_{Nano.}$ results (images) 10
Supplementary Figure 6 - TA-GAN input and output 11
Supplementary Figure 7 - TA-GAN$_{Syn.}$ morphological features 12
Supplementary Figure 8 - TA-GAN$_{Syn.}$ results (images) 13
Supplementary Figure 9 - TA-GAN$_{SA.}$ classification task 14
Supplementary Figure 10 - TA-GAN$_{Dend.}$ proportion of F-actin rings and fibers . 15
Supplementary Figure 11 - TA-CycleGAN domain adaptation 16
Supplementary Figure 12 - Segmentation results of U-Net$_{Live}$ and U-Net$_{Fixed}$ (Dice coefficient) 17
Supplementary Figure 13 - Segmentation results of U-Net$_{Live}$ and U-Net$_{Fixed}$ (ROC curves) 18
Supplementary Figure 14 - Imaging sequences; first and last images 19
Supplementary Figure 15 - Photobleaching from sequences of STED acquisitions 20
Supplementary Figure 16 - Live-cell imaging sequence using TA-GAN assistance, example 1 21
Supplementary Figure 17 - Live-cell imaging sequence using TA-GAN assistance, example 2 22
Supplementary Figure 18 - Evaluation of triggering methods 23
Supplementary Figure 19 - Reliability diagram for the segmentation of the *Translated F-actin dataset* 24
Supplementary Figure 20 - Live-cell imaging sequence using TA-GAN assistance, example 3 25
Supplementary Figure 21 - Registration of confocal and STED image pairs from the *Synaptic protein dataset* 26
Supplementary Figure 22 - Localization maps of synaptic protein clusters 27
Supplementary Figure 23 - Resolution measured with fitted profiles 28
Supplementary Figure 24 - TA-GAN$_{Live.}$ generation performance over imaging sequences 28
Supplementary Figure 25 - TA-GAN$_{Live.}$ generation performance as a function of the number of STED sub-regions 29

1 Supplementary tables

	Imaging parameters for the generation of the *Dendritic F-actin dataset* from (1)	Imaging parameters for the generation of the *Live F-actin dataset*
Fluorophore	Phalloidin-STAR635	SiR-Actin
Excitation power	6 µW	6.3 µW
Depletion power (at BFP)	180 mW	110 mW
Pixel Dwelltime	15 µs	5 µs
Line steps	4	8
Pixel size	20 nm	20 nm

Supplementary Table 1: Imaging parameters used for the live-cell experiments and compared with the parameters used in (1) for F-actin imaging in fixed neurons. The laser power was measured at the back focal plane.

	**Dataset**	**Input**	**Output**	**Task**	**Annotations**	**Task network**
TA-GAN *Ax.*	Axonal F-actin	Confocal (1 channel)	STED (1 channel)	Segmentation of axonal F-actin rings	Bounding boxes (1 class)	Not pretrained
TA-GAN *Nano.*	Simulated nanodomains	Simulated confocal (1 channel)	Simulated STED (1 channel)	Localization of nanodomains	Ground truth positions	Not pretrained
TA-GAN *Syn.*	Synaptic Proteins (localization)	Confocal (2 channels)	STED (2 channels)	Localization of synaptic protein cluster centroids	Wavelet detected centroids (2 channels)	Not pretrained
TA-GAN *Syn.*	Synaptic Proteins (segmentation)	Confocal (2 channels)	STED (2 channels)	Segmentation of protein clusters	Wavelet segmented clusters (2 channels)	Not pretrained
TA-GAN *SA.*	S. aureus (LR labels)	Bright-field (1 channel)	SIM (1 channel)	Segmentation of individual cells	Circular segmentation masks (1 class)	Not pretrained
TA-GAN *SA.*	S. aureus (HR labels)	Bright-field (1 channel)	SIM (1 channel)	Segmentation of cell division lines	Segmentation masks created from SIM images (1 class)	Not pretrained
TA-GAN *Dend.*	Dendritic F-actin	Confocal (1 channel)	STED (1 channel)	Semantic segmentation of dendritic F-actin rings and fibers	Bounding boxes (2 classes)	Not pretrained
TA-GAN *Live*	Live F-actin	Confocal + decision matrix + STED sub-region	STED (1 channel)	Semantic segmentation of dendritic F-actin rings and fibers	None	Pretrained and frozen

Supplementary Table 2: Summary of experimental settings using the TA-GAN for microscopy resolution enhancement.

**Hyperparam.**	**TA-GAN *Ax.***	**TA-GAN *Nano.***	**TA-GAN *Syn.***	**TA-GAN *SA.***	**TA-GAN *Dend.***	**TA-GAN *Live***
# train. images	377	1,080	690	202	4,331	753
# valid. images	56	120	35	64	659	47
Image size	224x224	64x64	512x512	256x256	224x224	Variable
Generator	9-blocks ResNet (2)					
Task net.	6-blocks ResNet					U-Net128 (3)
Discriminator	PatchGAN (4)					
Batch size	8	256	32	8	32	16
Learn. rate	0.0002					
Weight GAN	1					
Weight GEN	10	10	10	10	1	10
Data augm.	Horizontal and vertical flips, random crops, 90 degrees rotations					
Crop size	128	64	128	128	128	256
# epochs	1000	200	2000	2000	500	5000

Supplementary Table 3: Hyperparameters used to train the TA-GAN model on the six datasets presented.

	TA-GAN resolution [nm]	STED resolution [nm]
Axonal F-actin (far red)	75.3 ± 5.4	72.8 ± 4.6
Dendritic F-actin (far red)	60.5 ± 4.1	57.0 ± 3.5
Synaptic Proteins		
Bassoon (far red)	72.5 ± 2.8	67.3 ± 2.3
PSD95 (red)	87.2 ± 4.0	80.7 ± 3.6
Live F-actin (far red)	73.2 ± 6.3	69.3 ± 6.5

Supplementary Table 4: Resolution (mean ± standard error from the mean) approximated by measuring the Full Width at Half Maximum (FWHM) on fitted Lorentzian line profiles traced on random test images for each dataset (n=30 from 2 images for the *synaptic protein dataset*, n=20 from 5 images for the *Axonal F-actin dataset*, n=20 from 2 images for the *dendritic F-actin dataset*, n=20 from 3 images for the *Live F-actin dataset*). The corresponding resolution measured for confocal images (pixel size 20 nm) is 255.2 ± 1.9 nm for the far red channel (N = 21, 640 nm excitation) and 226.7 ±1.9 nm for the red channel (N = 34, 561 nm excitation).

Sub-region selection method	MSE	PSNR	SSIM	Sub-regions used out of 16
None	0.00762 ± 0.00440	21.73 ± 2.11	0.518 ± 0.123	N/A
Random	0.00696 ± 0.00411	22.17 ± 2.22	0.535 ± 0.124	9.9 ± 1.2
Min. intensity	0.00697 ± 0.00391	22.13 ± 2.17	0.534 ± 0.123	1.9 ± 0.9
Max. intensity	0.00676 ± 0.00398	22.16 ± 2.15	0.534 ± 0.125	2.6 ± 1.1
Normalized STD	0.00692 ± 0.00397	22.16 ± 2.15	0.536 ± 0.125	3.7 ± 1.5
SSIM	0.00676 ± 0.00392	22.27 ± 2.20	0.544 ± 0.127	3.2 ± 1.7
Optical Flow	0.00683 ± 0.00387	22.23 ± 2.20	0.542 ± 0.126	7.3 ± 1.7

Supplementary Table 5: Generation performance and diversity as a function of the method used to select the STED sub-region given as input to the generator. The metrics evaluated are the averaged Mean Squared Error (MSE), Structural Similarity Index Metric (SSIM), Peak Signal to Noise Ratio (PSNR), and the number of different STED sub-regions selected out of 16 over 15 frames (n=18 sequences). Similarity metrics are computed in the central 300 x 300 px ROI for n=168 pairs of synthetic and real STED images. Achieving a high diversity in the acquired STED sub-regions without compromising generation performance is essential to limit the photobleaching effects that would occur if using the same STED sub-region at each frame.

2 Supplementary figures

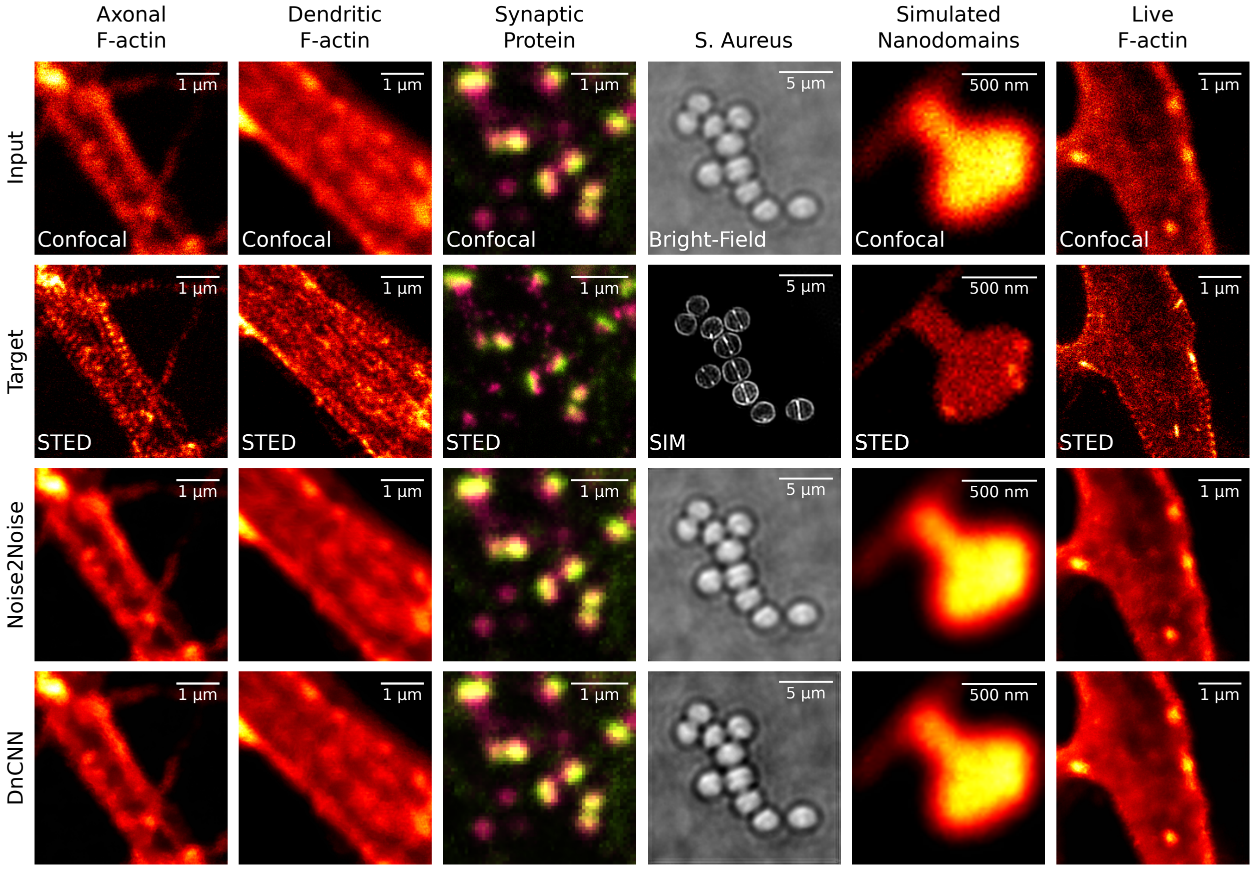

Supplementary Figure 1: The denoising approaches Noise2Noise (5) and Denoising CNN (6) cannot generate sub-diffraction structures of interest that are well resolved in the target modality (STED or SIM). Both models are directly applied using the trained weights from (7) since the training of those denoising approaches requires paired images with variable noise levels which were not available with our datasets. One representative image is chosen from the test set of each dataset (52 for the *Axonal F-actin dataset*, 26 for the *Dendritic F-actin dataset*, 9 for the *Synaptic protein dataset*, 5 for the *S. Aureus dataset*, 155 for the *Simulated nanoomain dataset*) and 109 for the *Live F-actin dataset*).

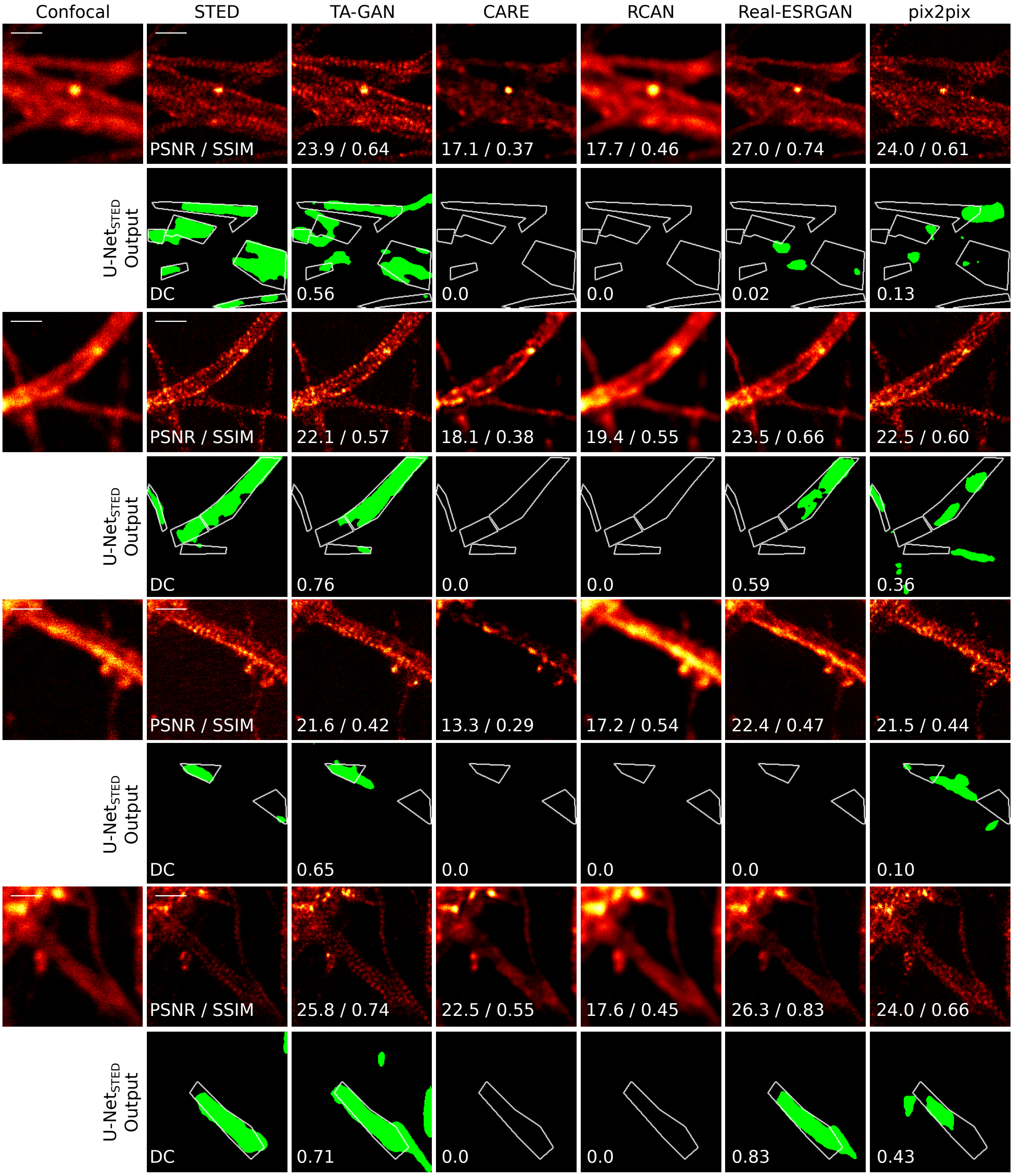

Supplementary Figure 2: Comparison of the TA-GAN with other resolution enhancement baselines on randomly selected examples out of the 52 images from the test set of the *Axonal F-actin dataset*. On all images from the test set, CARE (8) and RCAN (9) fail at generating axonal rings that are recognized by U-Net$_{Fixed-ax.}$. Real-ESRGAN (10; 11) and pix2pix (12) generate periodic F-actin nanostructures that are segmented by U-Net$_{Fixed-ax.}$ but are characterized by a significantly lower Dice Coefficient than TA-GAN$_{Ax.}$ when compared with the segmentation obtained on the corresponding real STED image (Extended data fig. 1). Scale bar: 1 µm. The intensity scaling of each image was normalized for display purpose by its own intensity maximum.

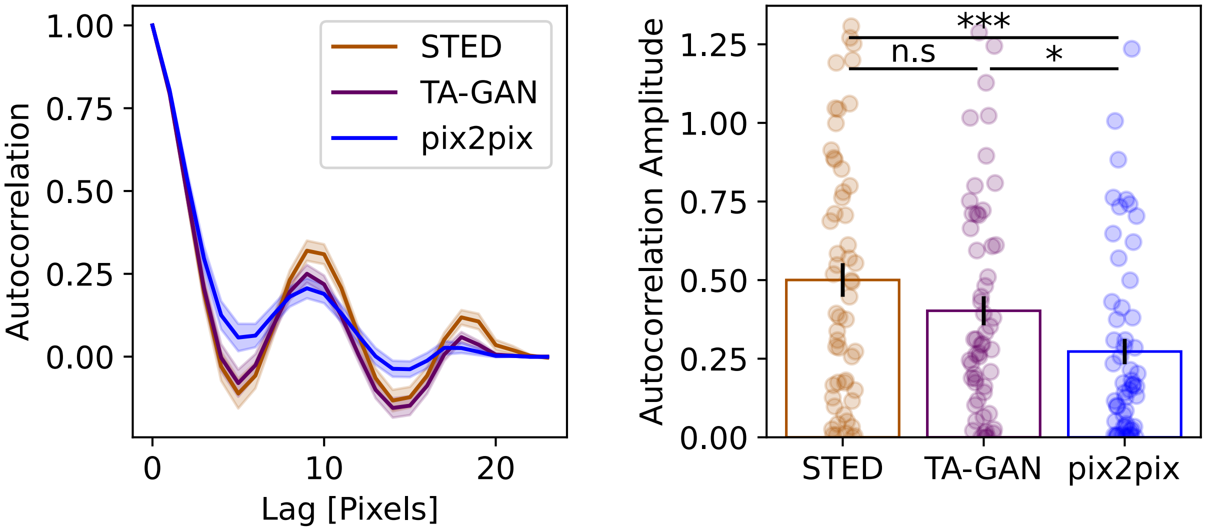

Supplementary Figure 3: Left, autocorrelation curve (13) (mean ± SEM) of axonal F-actin in real STED images, $\text{TA-GAN}_{Ax.}$ synthetic images, and pix2pix synthetic images. Right, measurement of the corresponding autocorrelation amplitude (mean ± SEM, n = 55 tracings from 26 test images from the *Axonal F-actin dataset*). Statistical analysis: two-sided Mann-Whitney U-test (14) for the null hypothesis that the distributions underlying the samples are identical: $p = 0.2$ for STED vs. TA-GAN, $p = 0.001$ for STED vs. pix2pix, $p = 0.02$ for TA-GAN vs. pix2pix ($***p < 0.001$, $*p < 0.05$, n.s. $p > 0.05$). The distributions are not drawn from a normal distribution based on the Shapiro-Wilk test for normality: $p = 3 \times 10^{-3}$ for STED, $p = 6 \times 10^{-4}$ for TA-GAN, $p = 2 \times 10^{-6}$ for pix2pix.

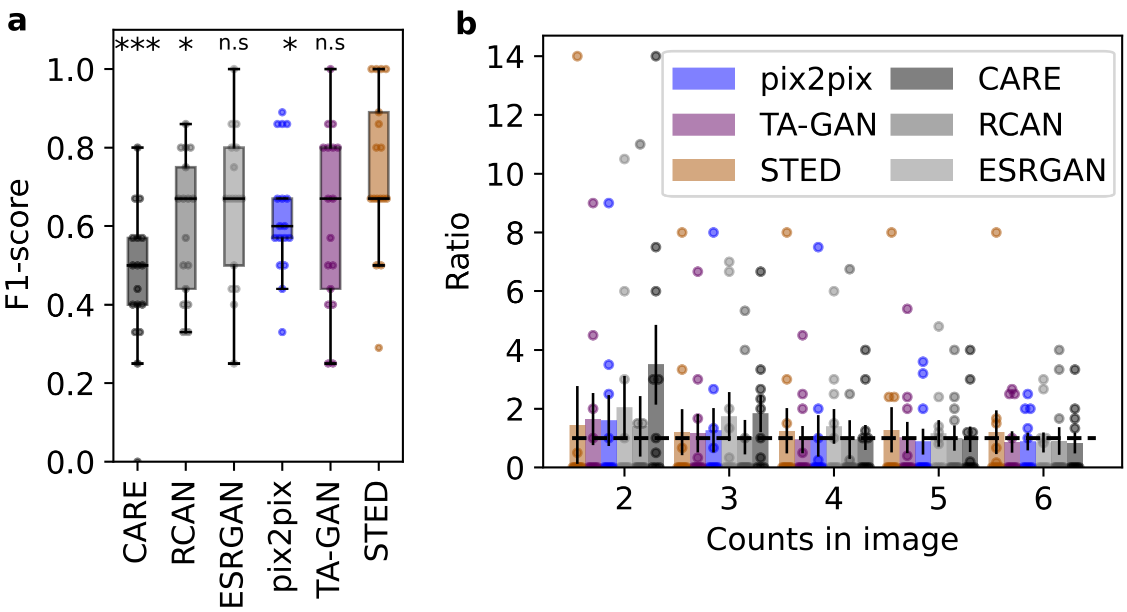

Supplementary Figure 4: a, F1-score for the localization of nanodomains on test images with two nanodomains spaced by less than 100 nm (n=17). Boxes extend from the lower to upper quartile values of each distribution, the line inside the box shows the median, and the whiskers extend to 1.5 times the interquartile range. Statistical analysis : two-sided Mann-Whitney U test (14) for the null hypothesis that the distributions underlying the results from each method differ from the STED distribution (CARE: $p = 6 \times 10^{-4}$, RCAN: $p = 0.05$, ESRGAN: $p = 0.2$, pix2pix: $p = 0.04$, TA-GAN: $p = 0.1$, $***p < 0.001$, $*p < 0.05$, n.s. $p > 0.05$). **b,** Ratio between the predicted and the ground truth number of nanodomains over the annotated two-nanodomains test set (n=75 images). Error bars show the standard error from the mean.

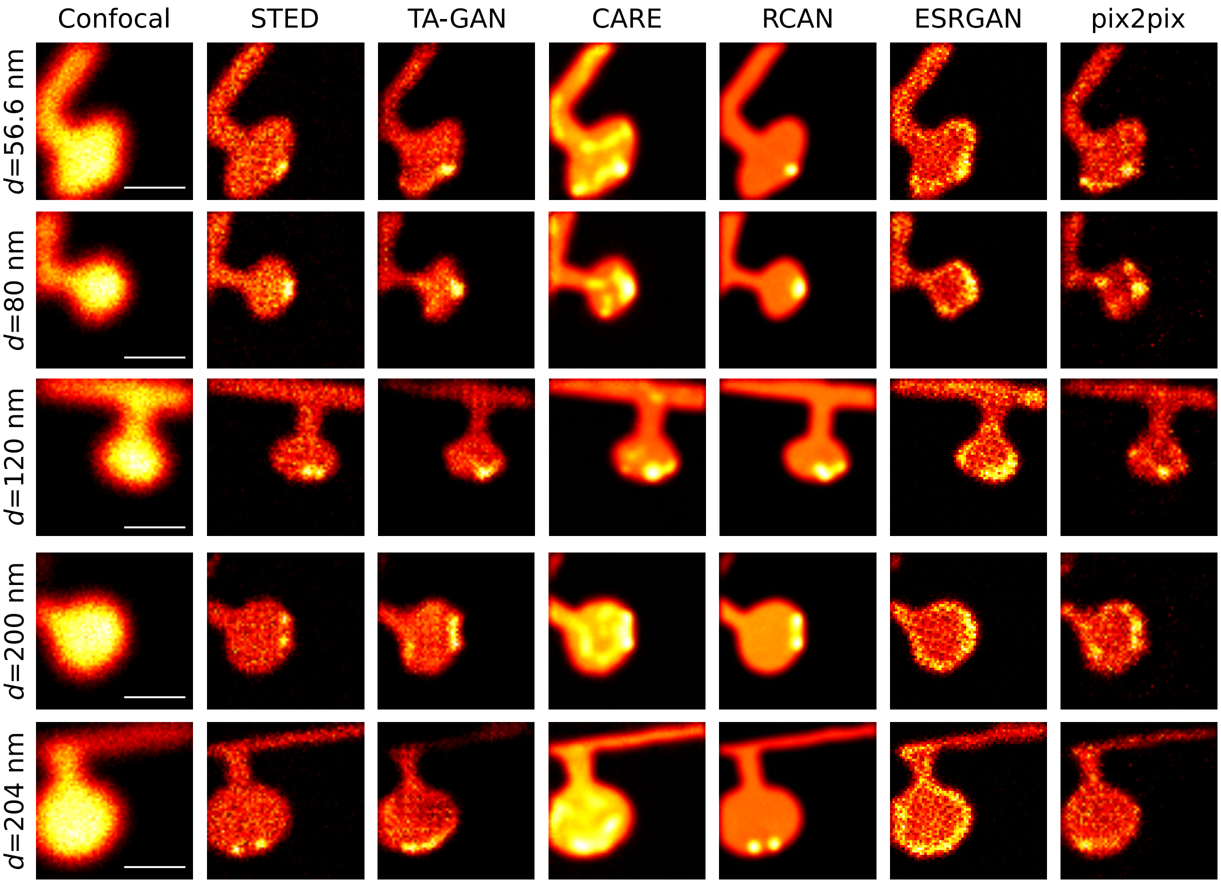

Supplementary Figure 5: Representative examples of synthetic images selected from the 80 images from the two-nanodomains *Simulated nanodomain dataset* test set. From top to bottom, the distance d between the pair of nanodomains in the ground truth datamap increases. Scale bar: 500 nm.

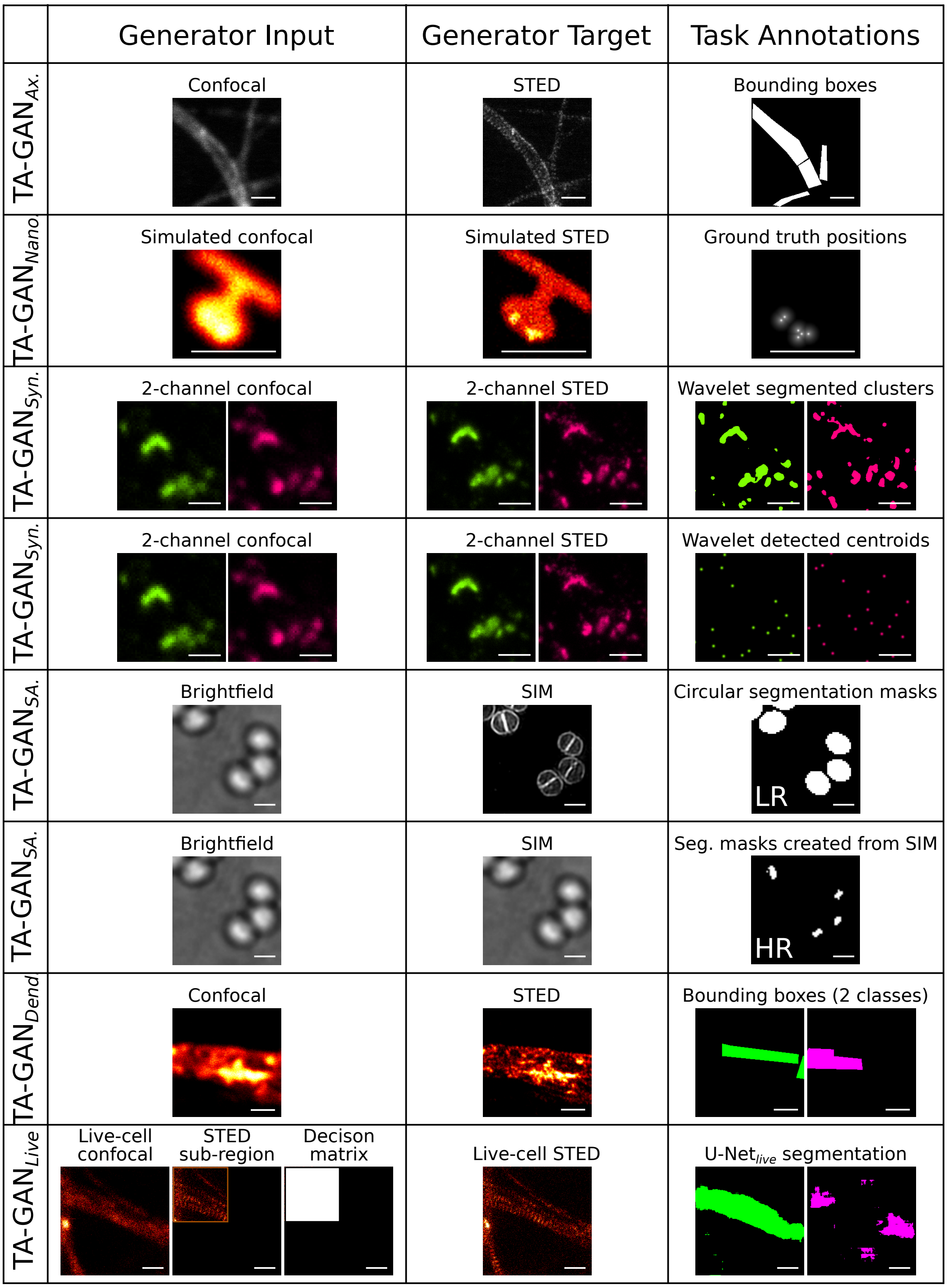

Supplementary Figure 6: Examples of input and target images for all TA-GAN models. Scale bars: 1 µm.

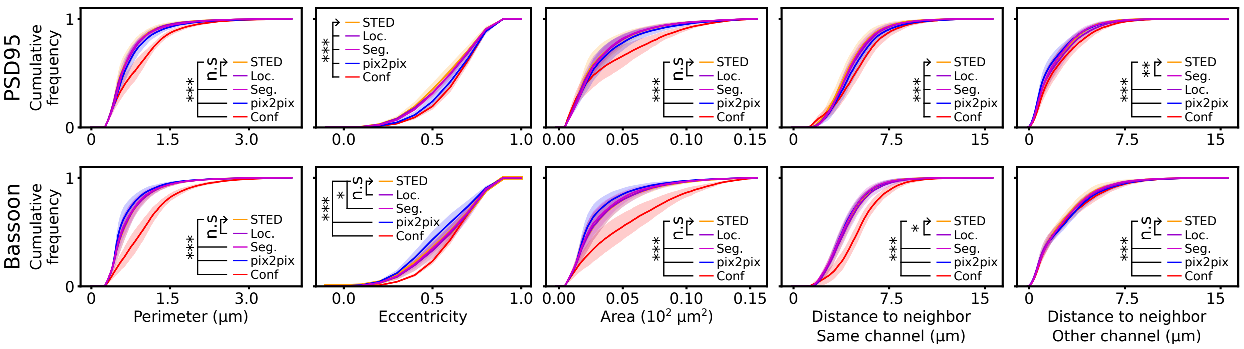

Supplementary Figure 7: Cumulative frequency plots of morphological features (perimeter, eccentricity, area, distance to nearest neighbor from the same channel and distance to nearest neighbor from the other channel) of coupled PSD95 and Bassoon clusters (15). Shown are images obtained with : *red* - confocal, *yellow* - STED, *blue* - pix2pix, *purple* - TA-GAN$_{Syn.}$ using a localization task, and *pink* - TA-GAN$_{Syn.}$ using a segmentation task. Shaded areas show the mean ± standard deviation from n = 9 independent images. The morphological features of the clusters generated by the TA-GAN$_{Syn.}$ trained with the localisation task show the highest similarity with the real STED images. Statistical analysis: two-sided two-sample Kolmogorov–Smirnov test for the null hypothesis that the continuous distribution underlying the results for each baseline is the same as the one underlying the STED results ($*** \ p < 0.001$, $** \ p < 0.01$, $*p < 0.05$, n.s. $p > 0.05$).

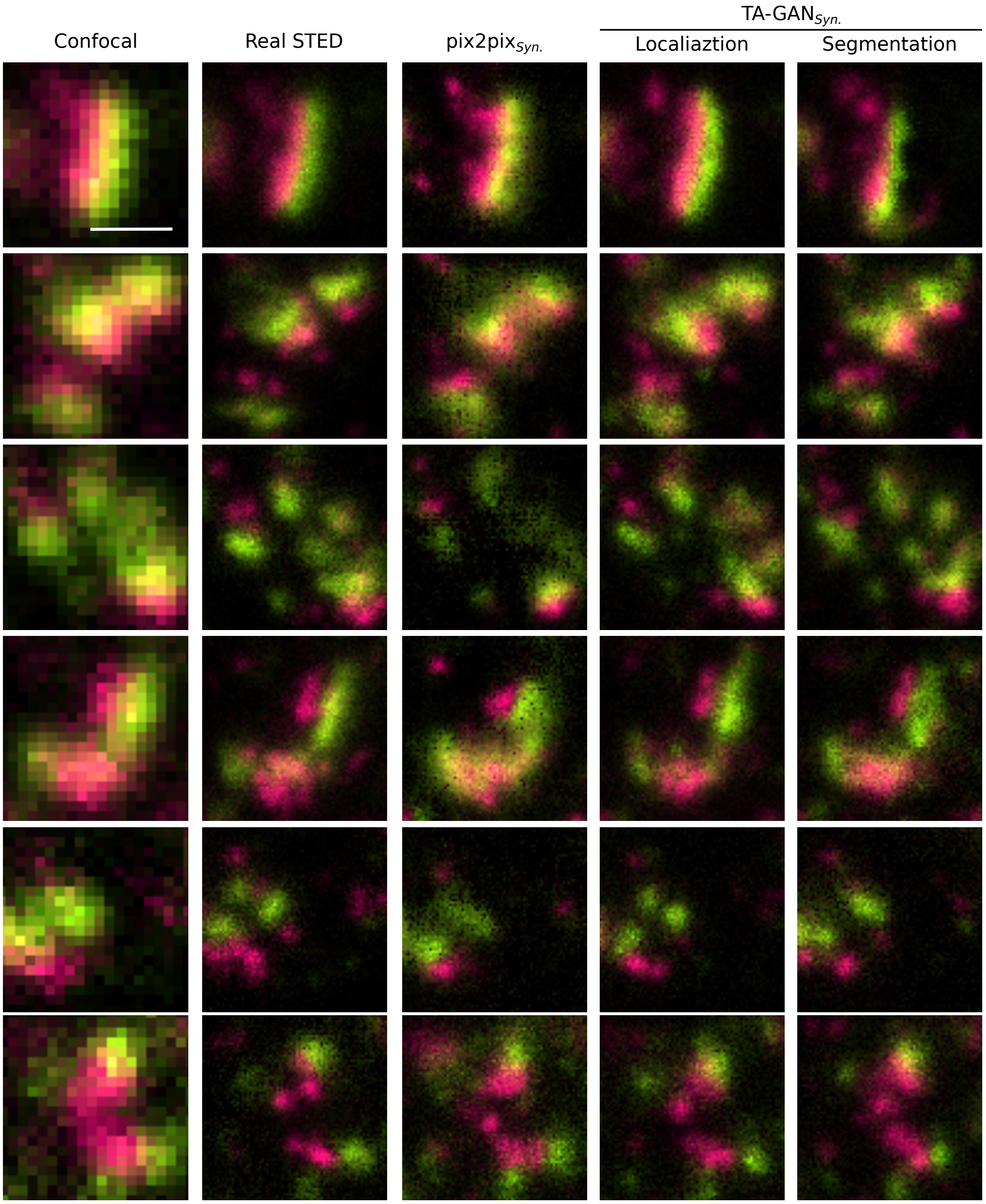

Supplementary Figure 8: a, Example of synthetic images generated by the TA-GAN$_{Syn.}$ on the test set of the *Synaptic protein dataset.* Each crop is normalized to its own maximum for better visualization of dim clusters. The crops were selected as representative examples out of the 9 large images from the test set. Scale bar: 500 nm.

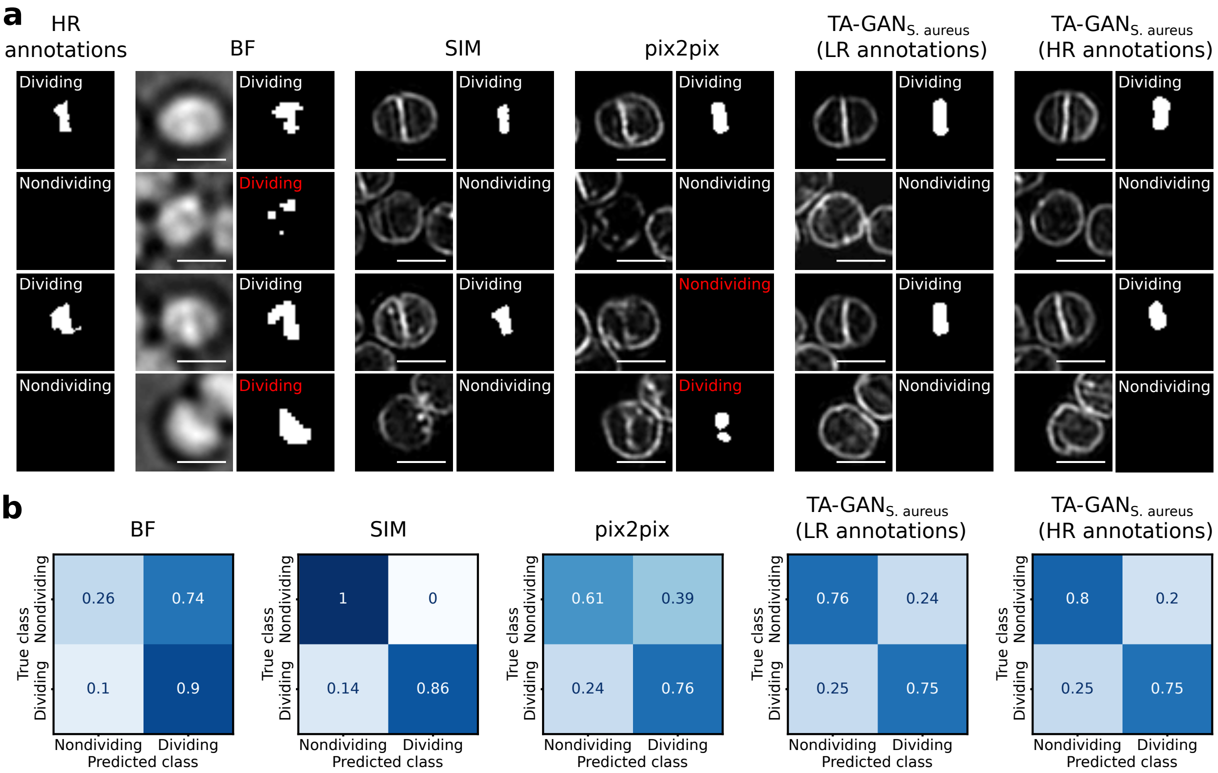

Supplementary Figure 9: Classification task developed for the evaluation of TA-GAN$_{SA.}$. **a,** Single-cell crops and their corresponding annotations selected as representative examples from the 410 cells of the test set. The ground truth annotations are obtained from the high resolution (HR) SIM images. The threshold-based segmentation of the BF images does not allow to reliably identify dividing cell boundaries. The ResNet$_{SA.}$ predicts reliable segmentation masks for dividing cell boundaries on the SIM images. The ResNet$_{SA.}$ is applied for the segmentation of dividing cell boundaries on synthetic SIM images generated with pix2pix$_{SA.}$ and with the TA-GAN$_{SA.}$ trained either with whole-cell low-resolution (LR) annotations (16) or the ground truth HR annotations shown in the first column. The ResNet$_{SA.}$ trained on real images of the *S. aureus dataset* predicts reliable segmentation masks of the dividing cell boundaries on the synthetic images generated with both training schemes of the TA-GAN$_{SA.}$, but fails at segmenting the structures on the images generated with pix2pix. The class is predicted by applying a threshold on the number of segmented pixels (Methods). All images are normalized to their own maximum. Scale bars: 1 µm. **b,** Confusion matrices for the classification of dividing or non-dividing cells. Classification accuracy : BF 51.0%, SIM 94.4%, pix2pix 66.9%, TA-GAN$_{SA.}$ trained with LR annotations 75.9%, TA-GAN$_{SA.}$ trained with HR annotations 78.3%.

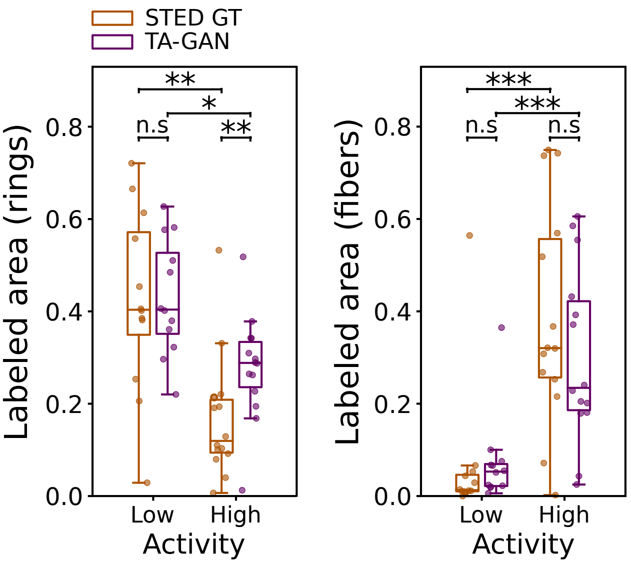

Supplementary Figure 10: The proportion of dendritic F-actin rings (left) is significantly larger at low neuronal activity (5mM Mg^{2+}, 0.6mM Ca^{2+} blocking solution) compared to high activity (0Mg^{2+}/Gly/2.4mM Ca^{2+}) in both real (orange) and TA-GAN$_{Dend.}$ generated synthetic (purple) STED images (STED: $p = 0.0007$, TA-GAN$_{Dend.}$: $p = 0.004$). The opposite is observed for F-Actin fibers (right) for both the real ($p = 0.0006$) and the synthetic STED images ($p = 0.0009$). Boxes extend from the lower to upper quartile values of each distribution, the line inside the box shows the median, and the whiskers extend to 1.5 times the interquartile range. Statistical analysis: two-sided Mann-Whitney U test (14) for the null hypothesis that the distributions underlying the compared data are different ($*** \ p < 0.001$, $** \ p < 0.01$, $*p < 0.05$, n.s. $p > 0.05$). n=12 independent images for low activity and 14 independent images for high activity.

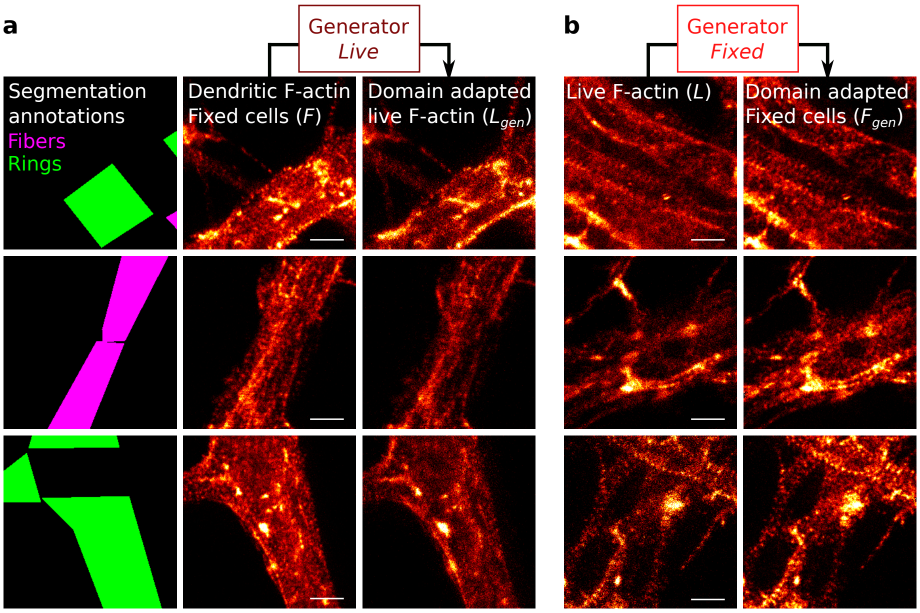

Supplementary Figure 11: Fixed-cell to live-cell imaging domain adaptation. **a,** Real STED images of fixed cells are translated to synthetic images from the live-cell imaging domain by Generator$_{Fixed}$. Following the image translation, the manual segmentation masks associated with the real STED images of fixed cells are associated to the synthetic live-cell images. **b,** Real live-cell images of F-actin are translated to the fixed-cell imaging domain with Generator$_{Live}$. Examples were randomly selected from the 4331 training images of the *Dendritic F-acitn dataset* and the 753 training images from the *Live F-actin* dataset. Scale bars: 1 µm.

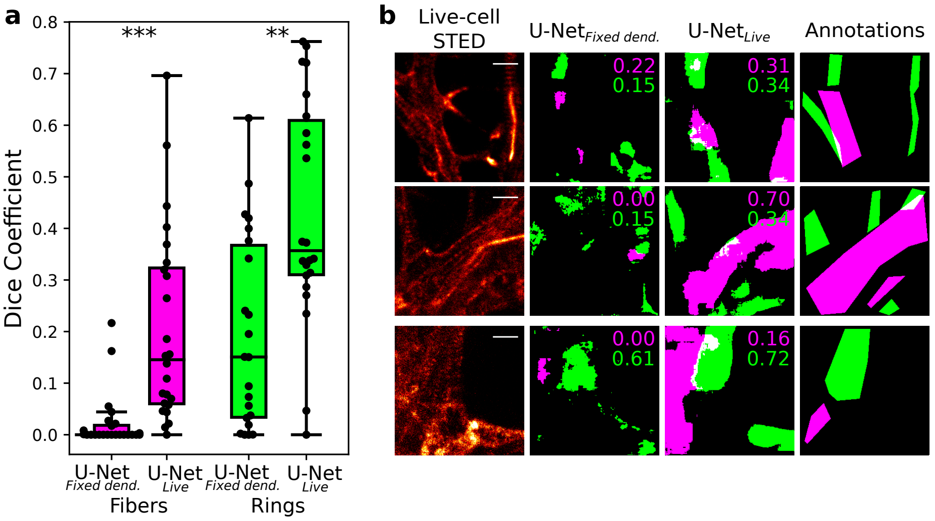

Supplementary Figure 12: Measured dice coefficient (DC) for the segmentation of F-actin rings and fibers in real live-cell STED images. **a,** The DC is computed between the predictions of the U-Net segmentation networks and manual polygonal bounding box on live-cell STED images of size (28 images of size 256 x 256 pixels, 5.12 x 5.12 µm.) that were annotated by an expert. 22 images contained F-actin rings and 24 images F-actin fibers. The DC is significantly improved for the segmentation of F-actin nanostructures on live-cell STED images with U-Net$_{Live}$, trained with synthetic images translated to the live-cell domain from the *Translated F-actin dataset*(L', mean $DC_{fibers} = 0.20$, $DC_{rings} = 0.43$), in comparison with the U-Net$_{Fixed}$, a U-Net trained with the same original fixed-cells STED images from the *Dendritic F-actin dataset* (F, mean $DC_{fibers} = 0.02$, $DC_{rings} = 0.20$). Boxes extend from the lower to upper quartile values of each distribution, the line inside the box shows the median, and the whiskers extend to 1.5 times the interquartile range. Statistical analysis: two-sided two-sample Kolmogorov–Smirnov test for the null hypothesis that the distributions underlying the samples are identical ($***p = 1 \times 10^{-6}$, $**p = 0.003$). **b,** Examples of predicted segmentation masks from the *Live F-actin dataset* test set with their individual DC written in the top right corner for F-actin fibers (magenta) and rings (green). From left to right: live-cell STED image, predicted segmentation from the U-Net$_{Fixed}$ trained on fixed-cell images (F), predicted segmentation from U-Net$_{Live}$ (L', and manual expert annotations. Top row: best DC on fibers obtained with U-Net$_{Fixed}$. Middle row: best DC on fibers obtained with U-Net$_{Live}$. Bottom row: best DC on rings obtained with U-Net$_{Fixed}$ out of 28 annotated test images. Scale bars: 1 µm.

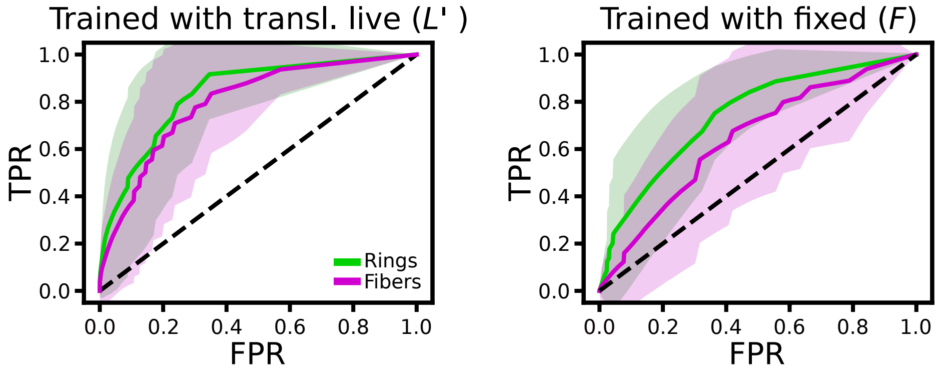

Supplementary Figure 13: ROC curves computed over the test set (n=28) of synthetic images from the live-cell domain from the live-cell domain, using the manual annotations as ground truth. The TA-CycleGAN translates real fixed-cell STED images from the *Dendritic F-actin dataset* into corresponding synthetic images from the live-cell STED imaging domain (*Translated F-actin dataset*). Left: the U-Net$_{Live}$ is trained on the *Translated F-actin dataset* (L'). On the right: U-Net$_{Fixed}$ is trained on the *Dendritic F-actin dataset* (F). The results are reported for F-actin fibers (magenta) and rings (green). The shaded region corresponds to the mean $\pm$ standard deviation. Dashed line represents the worst achievable performance (random classifier, AUROC=0.5).

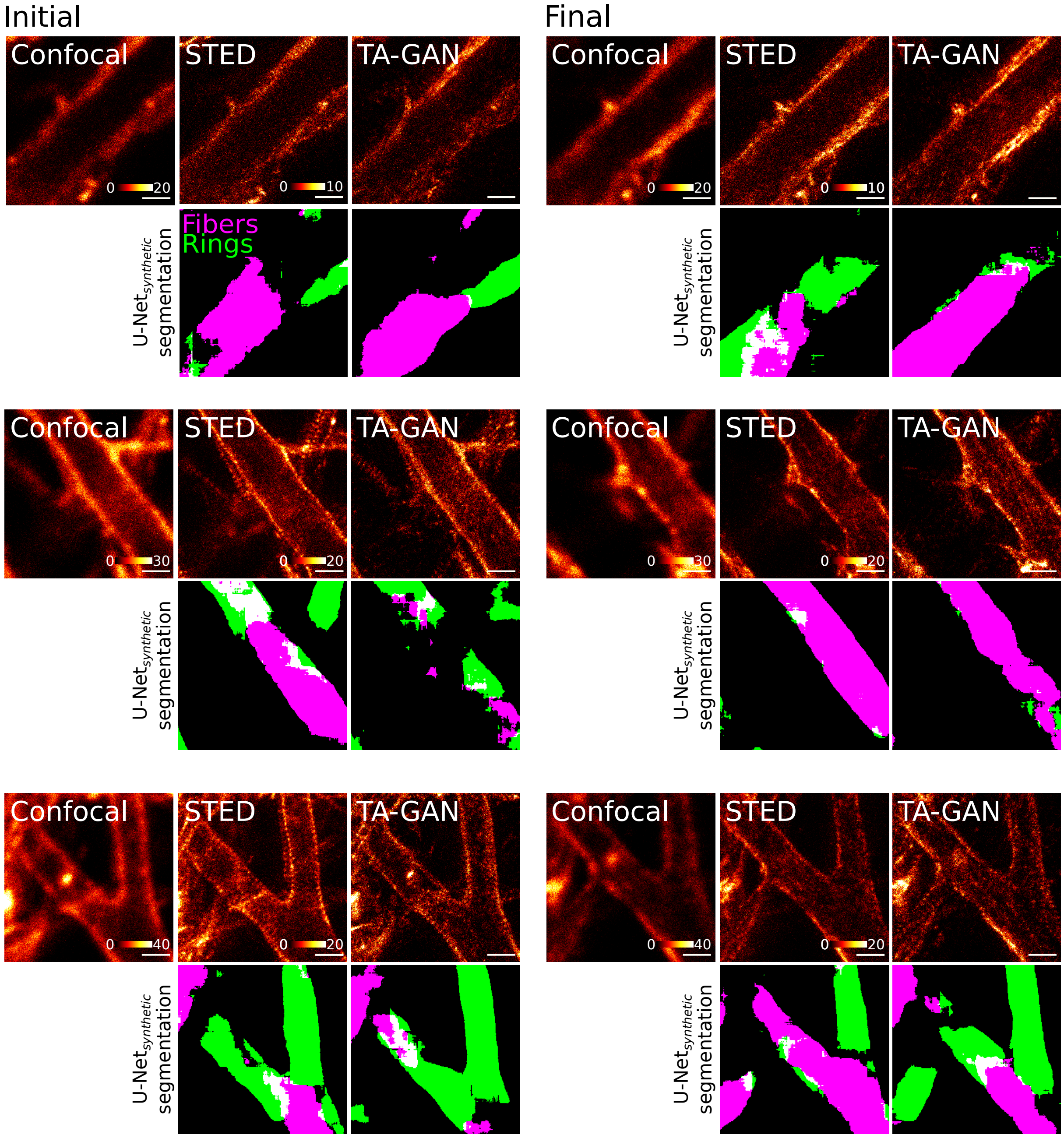

Supplementary Figure 14: Live-cell imaging sequences using the TA-GAN$_{Live}$ before (left, *initial*) and after (right, *final*) neuronal stimulation. Images were selected out of 159 imaging sequences as representative examples of the TA-GAN$_{Live}$ performance. Scale bars: 1 µm.

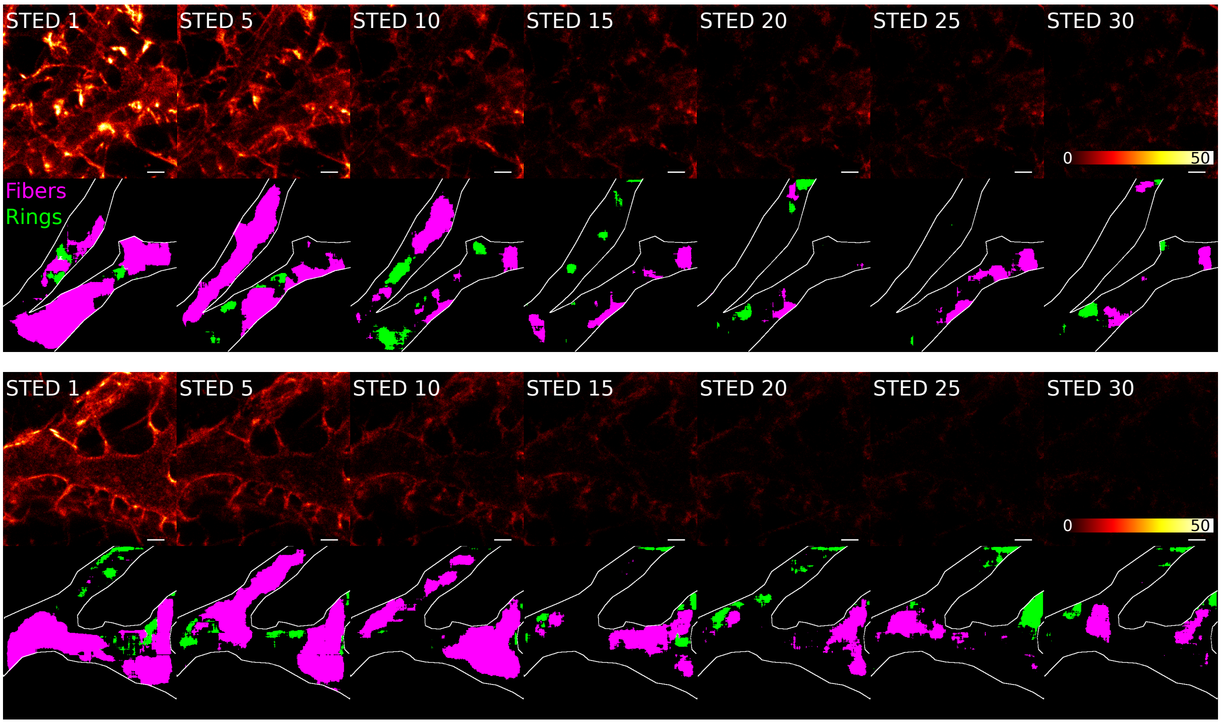

Supplementary Figure 15: Two sequences of 30 STED acquisitions are used to evaluate the photobleaching. The STED images at frames 1, 5, 10, 15, 20, 25, and 30 are displayed using the same intensity scale. The segmentation prediction is obtained with the U-Net$_{Live}$. The signal in the STED images quickly degrades after a few acquisitions, and U-Net$_{Live}$ fails at accurately identifying the F-actin nanostructures. The example was randomly selected out of 45 series of STED acquisitions. Scale bars: 1 µm.

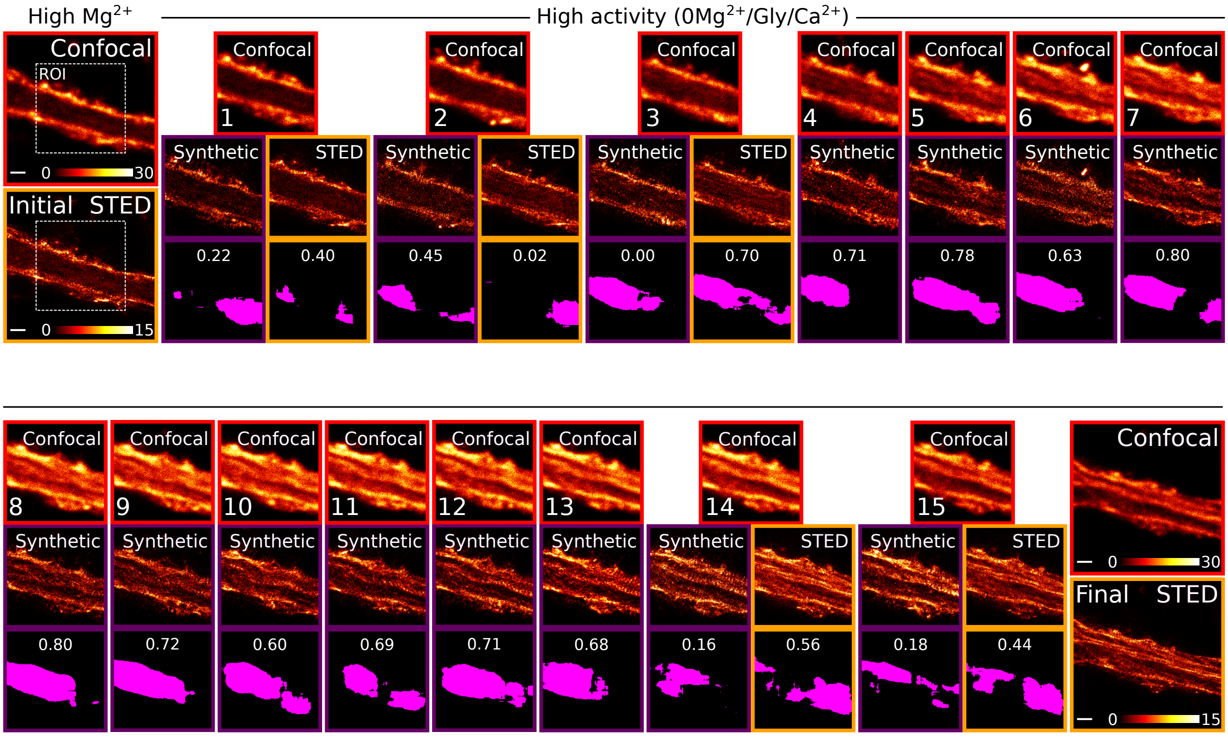

Supplementary Figure 16: Images acquired at all time points for the time series shown in Figure 4**b**. First row shows the confocal images (red), second row shows the STED images which are either synthetic (purple) or real (orange), third row shows the segmentation of fibers by applying the U-Net$_{Live}$ on the STED (synthetic or real) images. The DC computed with the segmentation of the most recent real reference STED as the ground truth is displayed on each segmentation map. The intensity scale is constant for all confocal images (0-30 counts) and for all STED images (0-15 counts). The series was chosen as a representative example of fibers remodeling from a total of 72 series using this acquisition scheme. Scale bars: 1 µm.

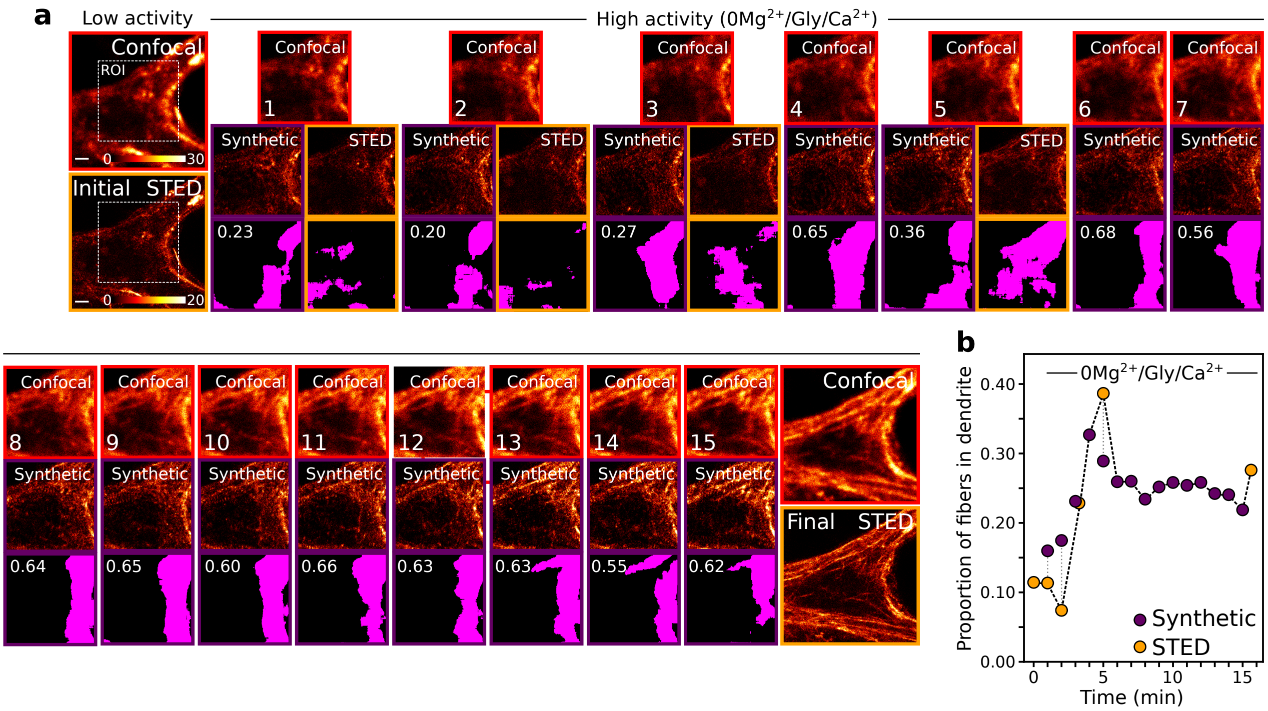

Supplementary Figure 17: Additional example showing the prediction of F-actin remodelling with TA-GAN$_{Live}$. **a,** Live-cell imaging of dendritic F-actin before (initial), during (frames 1-15) and after (final) application of a $0Mg^{2+}$/Gly/2.4mM Ca^{2+} solution, which promotes synaptic NMDA receptor activity. Shown are the confocal images (red, top row), synthetic STED images (purple, middle row), and real STED images when acquired (orange, middle row), and corresponding segmentation masks for F-actin fibers (bottom row). **b,** The U-Net$_{Live}$ is used to compute the proportion of dendritic F-actin fibers on either the real STED (orange) or the synthetic STED (purple). In this specific case, STED acquisitions are triggered after 1, 2, 3, and 5 minutes, allowing to image in nanoscale resolution the increase in the proportion of F-actin fibers. The proportion of F-actin fibers remains stable for the following time points (6-15) and no STED images are triggered. The series was chosen as a representative example of fibers remodeling from a total of 72 series using this acquisition scheme. Scale bars: 1 μm.

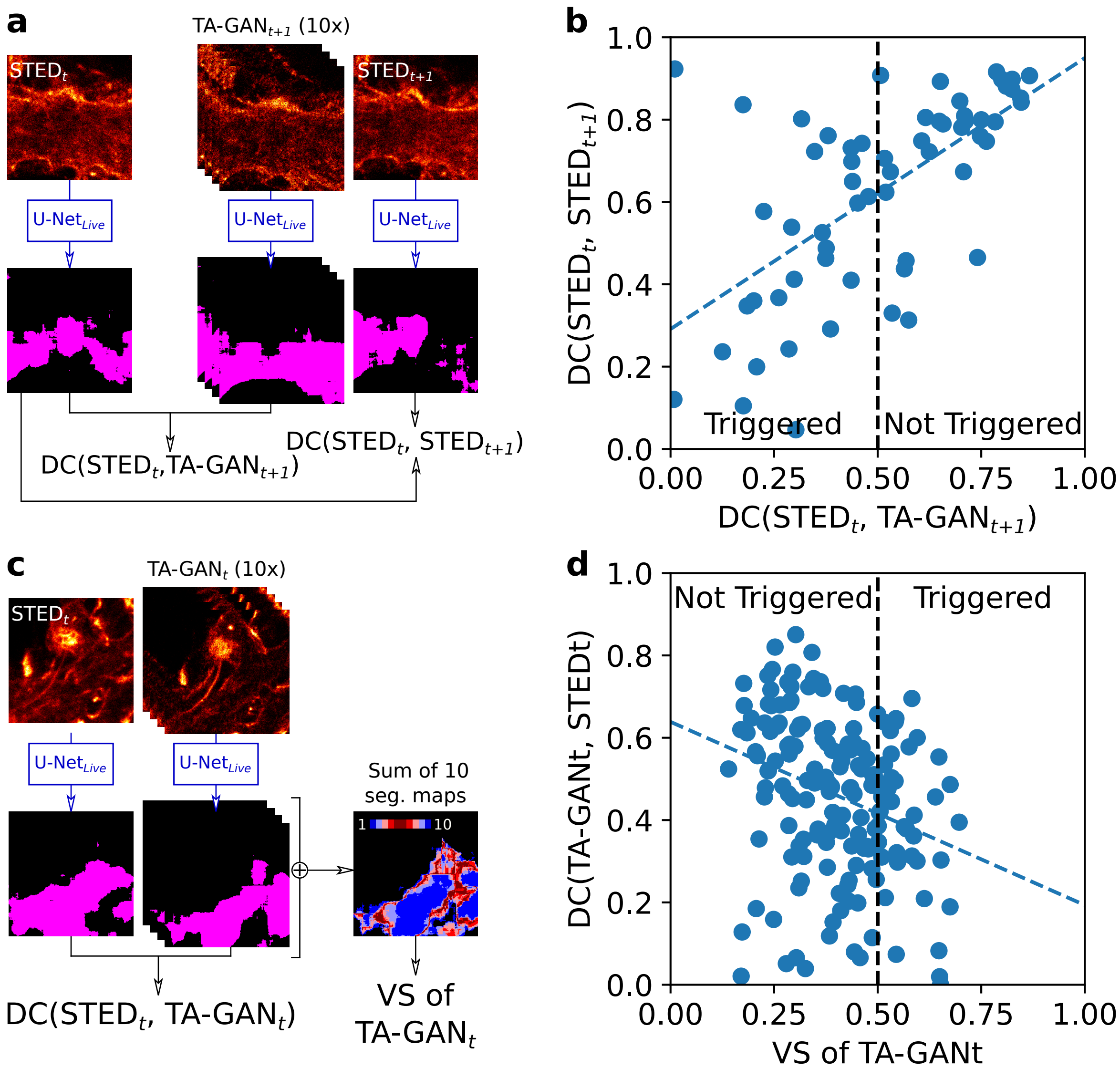

Supplementary Figure 18: (Caption next page.)

Supplementary Figure 18: (Previous page.) **a,** The Dice coefficient (DC) between the real STED at time t and the TA-GAN synthetic STED at time $t+1$ (one minute later) is computed. The DC between the real STED from time t and the next STED from time $t+1$ is computed. **b,** Both DC computed in **a** are compared. The change in the segmentation between the two real STED images at times t and $t+1$ (y-axis) is positively correlated with the similarity between the real STED image at time t and the synthetic STED image at time $t+1$ (x-axis, $r^2 = 0.445$, $p = 6 \times 10^{-9}$, two-sided test for the null hypothesis that the slope is zero, using Wald Test with t-distribution of the test statistic). The correlation implies that the DC threshold can be adjusted to the objective of a given experiment: for example increasing the threshold would trigger less acquisition of STED images and only at frames where the amount of change is higher. **c,** The DC between the real and synthetic STED images at time t is computed. The segmentation maps of the 10 synthetic STED images are used to compute the variability score (VS) at time t. **d,** The DC and the VS computed in **c,** are compared. The changes between the segmentation of the real and synthetic STED images at time t (y-axis) is negatively correlated with the variability score (x-axis, $r^2 = 0.0876$, $p = 1 \times 10^{-4}$, two-sided test for the null hypothesis that the slope is zero, using Wald Test with t-distribution of the test statistic). The correlation implies that the VS measures the uncertainty of the TA-GAN, since higher VS correlates with higher disparity with the real STED segmentation. Statistical analysis : Wald Test with t-distribution of the test statistic for the null hypothesis that the correlation is zero. The analysis was performed on control sequences of two frames with neuronal stimulation (n=60) for which the confocal and STED images were always acquired at time steps t and $t+1$.

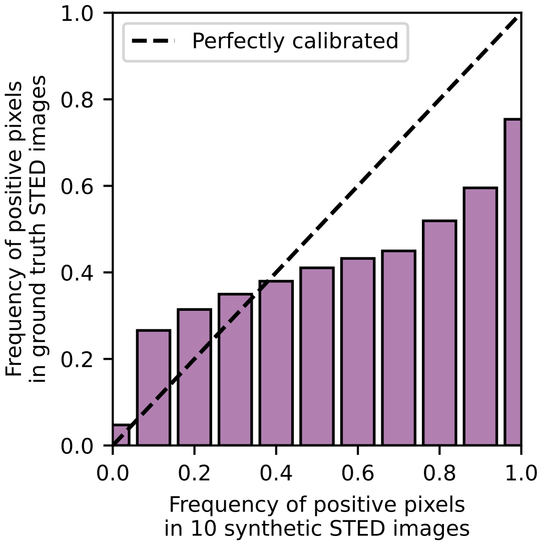

Supplementary Figure 19: Reliability diagram for the segmentation of F-actin fibers on the *Translated F-actin dataset*. Positive pixels are pixels identified as fibers by U-Net$_{Live}$. Predicted probability (x-axis) is obtained from the segmentation of 10 synthetic STED images generated by TA-GAN$_{Live}$. Observed probability (y-axis) is obtained from the segmentation of the corresponding ground truth STED images. n=162 ROIs of size 300 x 300 pixels. The fact that the function is monotonic validates the use of this metric to trigger a STED acquisition.

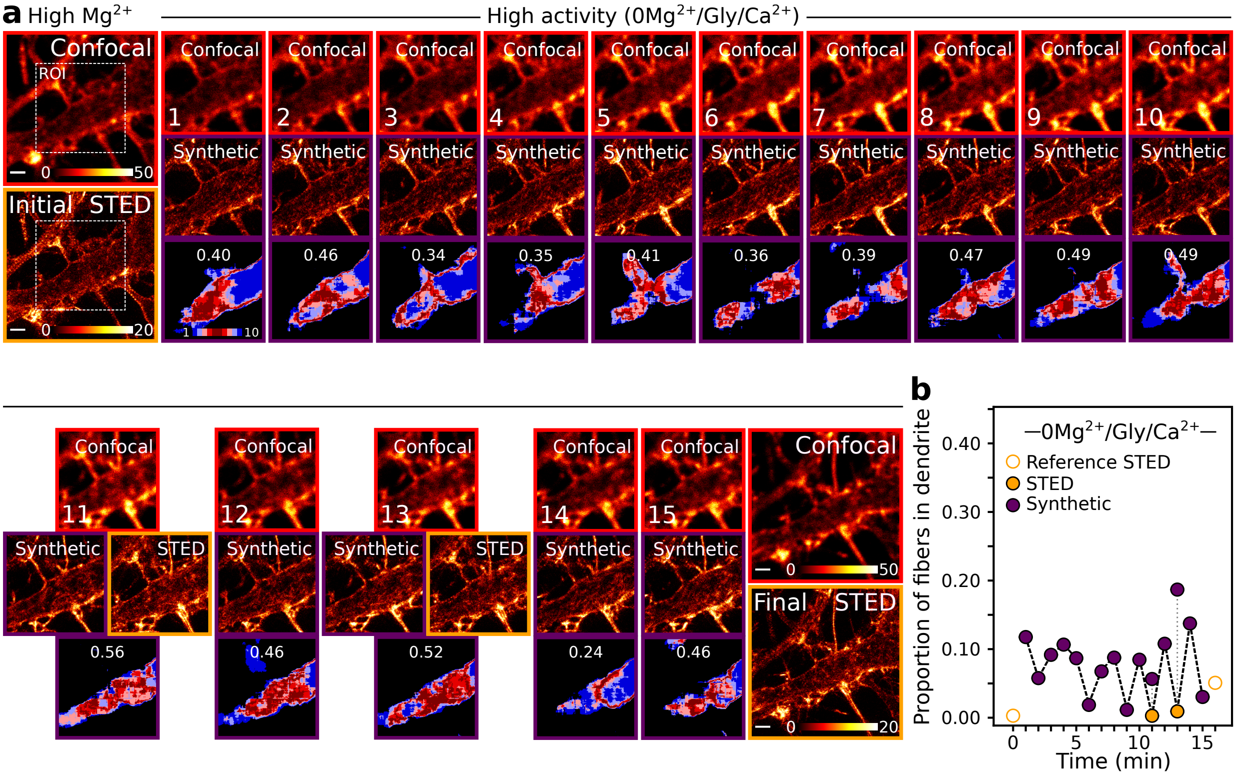

Supplementary Figure 20: **a,** Images at all time points for the time series shown in Figure 5**a**. First row shows the confocal images (red), second row shows the STED images which are either synthetic (purple) or real (orange), third row shows the sum of 10 segmentation maps of F-actin fibers obtained by applying the U-Net$_{Live}$ the STED images. Blue pixels have low variability and red pixels have high variability. The the variability scores are displayed on each segmentation maps. The intensity scale is constant for all confocal images (0-50 counts) and for all STED images (0-20 counts). Scale bars: 1 µm. **b,** Proportion of fibers on the dendrite on either the real STED (orange) or the synthetic STED (purple) images. Initial and final reference STED images (empty orange bullets) are acquired at each round. This series was chosen as a representative example where there is no activity-dependent increase from a total of 87 series using this acquisition scheme.

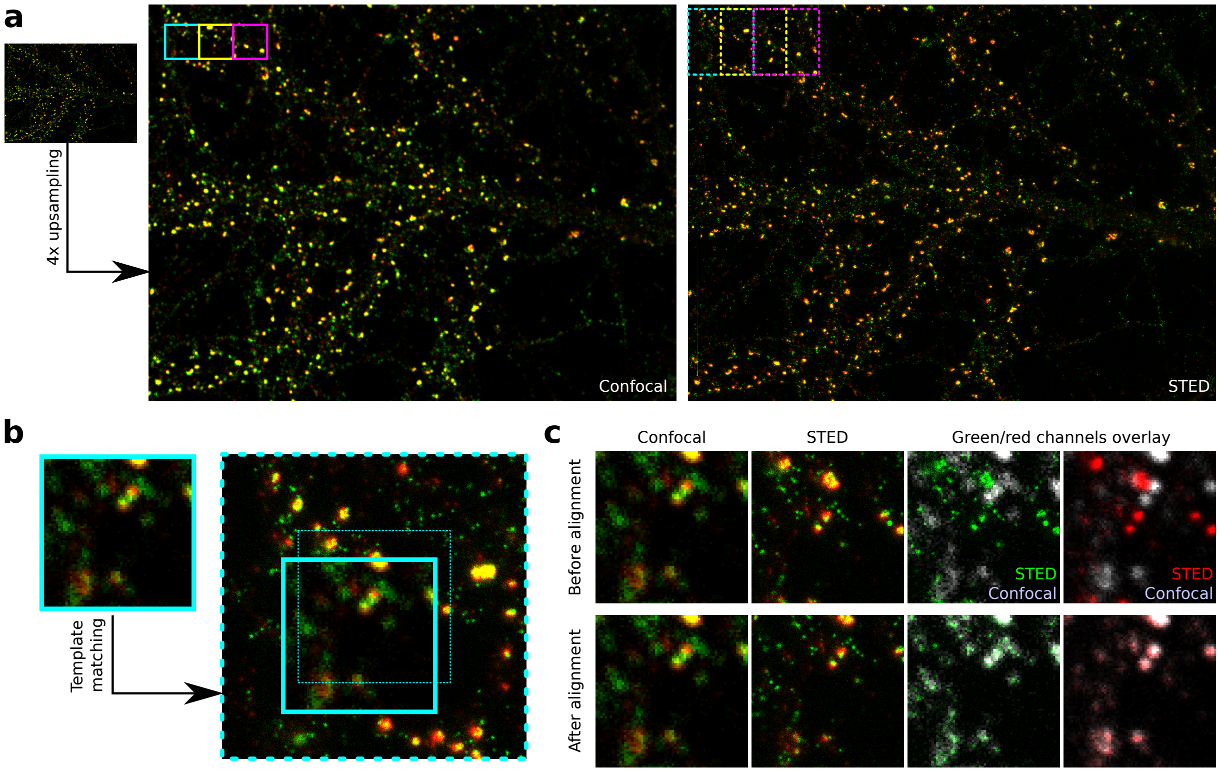

Supplementary Figure 21: Registration method to align confocal and STED image pairs from the *Synaptic protein dataset*. **a,** The confocal image is upsampled with nearest-neighbor interpolation to match the size of the STED image. The three regions identified (green, yellow and magenta squares) show the sliding window method used to iteratively choose sections to match. **b,** For each 512 x 512 px square region of the STED image, the centered 256 x 256 px square region from the confocal image is matched using the *OpenCV* (17) 3.1.0 template matching library. The dashed square identifies the center of the region, and the full line identifies the region which matches the confocal crop. **c**) The confocal crop, the STED corresponding crop, and a channel-wise overlay of the two modalities before (top) and after (bottom) alignment.

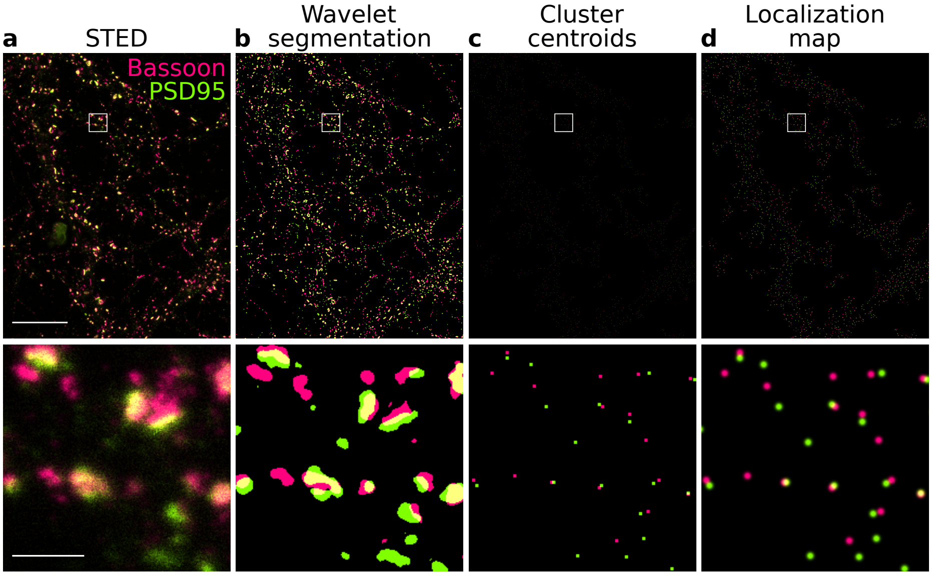

Supplementary Figure 22: Localization maps of synaptic protein clusters. **a,** Two-color STED image of PSD95-Alexa594 (green) and Bassoon-STAR635 (magenta) in fixed neurons (18). **b,** Wavelet segmentation of Bassoon (pink) and PSD95 (green) clusters. **c,** Weighted centroids obtained from the segmentation of individual clusters. **d,** Final localization maps generated by applying a Gaussian filter on the weighted centroids (Methods). Scale bars: 10 µm (top), 1 µm (bottom).

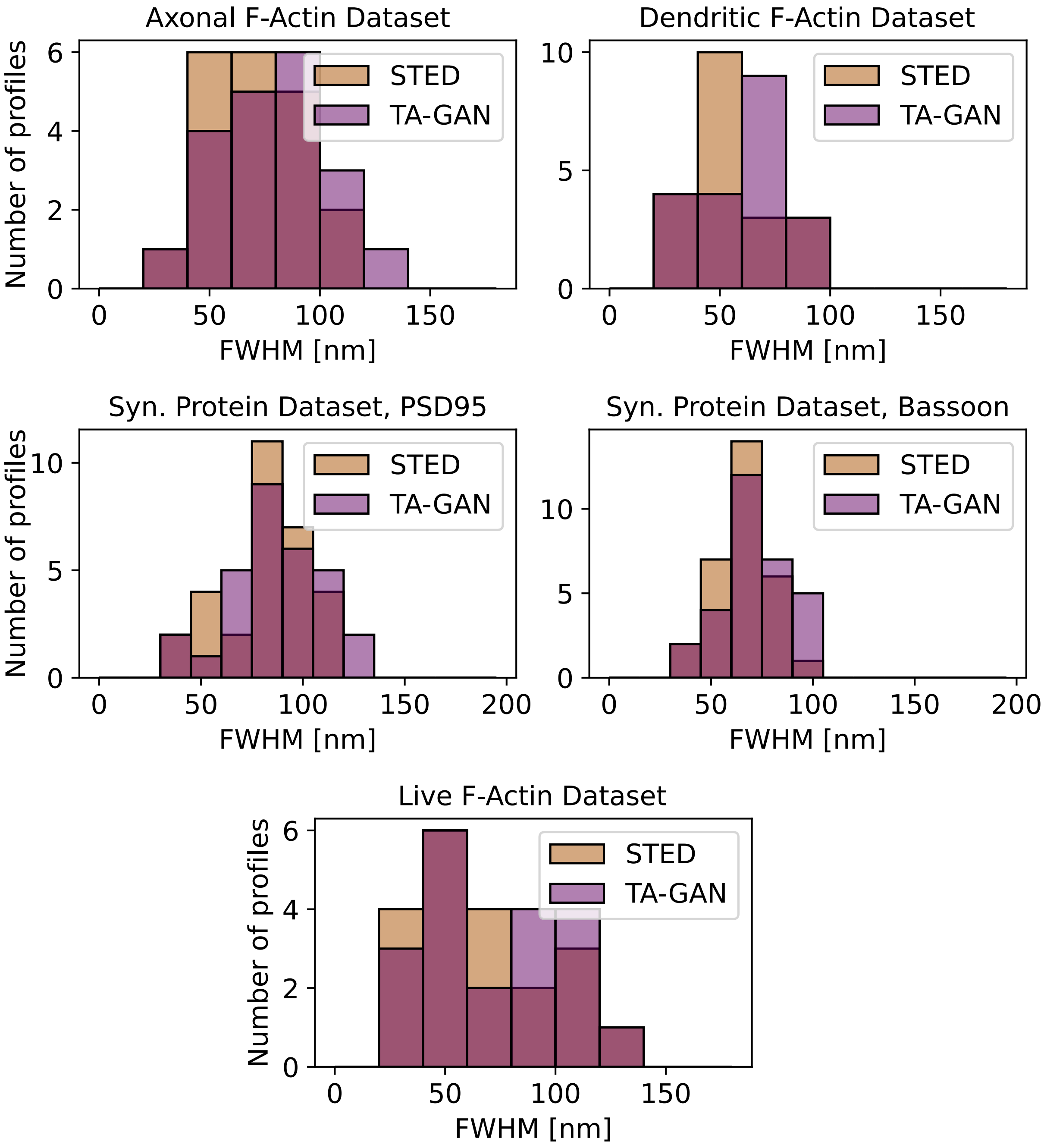

Supplementary Figure 23: Distribution of the measured Full Width at Half Maximum (FWHM) on fitted Lorentzian line profiles traced on randomly chosen test images for each dataset (n=30 from 2 images for the *Synaptic protein dataset*, n=20 from 5 images for the *Axonal F-actin dataset*, n=20 from 2 images for the *Dendritic F-actin dataset*). TA-GAN and STED distributions are not statistically different for all datasets (p>0.05). Statistical analysis : null hypothesis that the distributions are identical is verified with a two-sided Mann-Withney U-test for the *dendritic F-actin dataset* ($p = 0.31$) and the *Live F-actin dataset* ($p = 0.68$) (distributions are not drawn from a normal distribution based on the Shapiro-Wilk test for normality), and a two-sided independent 2 sample t-test for the *Axonal F-actin dataset* ($p = 0.73$) and the *Synaptic Proteins dataset* ($p = 0.24$ for PSD95, $p = 0.16$ for Bassoon) (equality of variances is verified by the F-test with a significance level of 5%). All bin widths correspond to the pixel size.

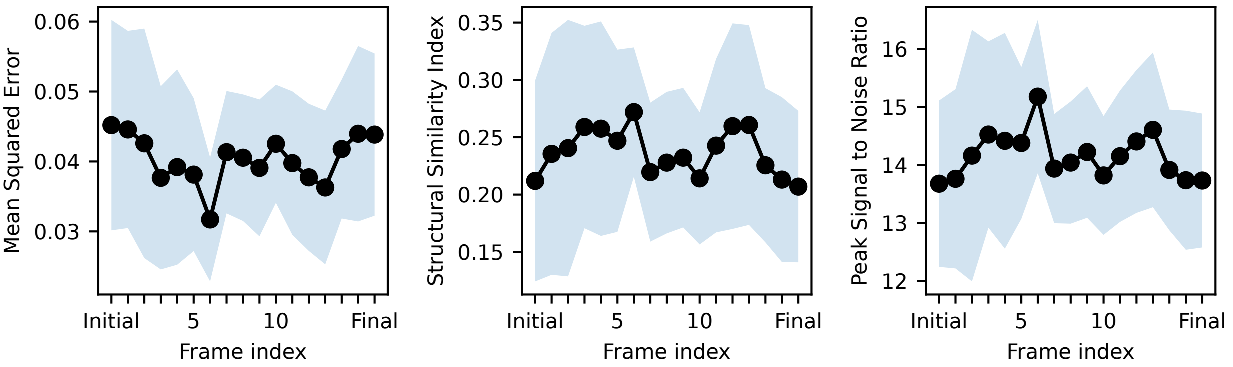

Supplementary Figure 24: Comparison between the TA-GAN$_{live}$ synthetic STED images and the corresponding ground truth STED images using the mean squared error, peak signal to noise ratio, and structural similarity). The images (synthetic and STED) are normalized prior to computing the metrics. For the three metrics, the median of the distributions are not statistically different. Solid lines shows the mean value at each frame and the shaded area corresponds to the standard deviation. Statistical analysis : Kruskal-Wallis H-test for independent samples (distributions are not drawn from a normal distribution based on the Shapiro-Wilk test for normality).

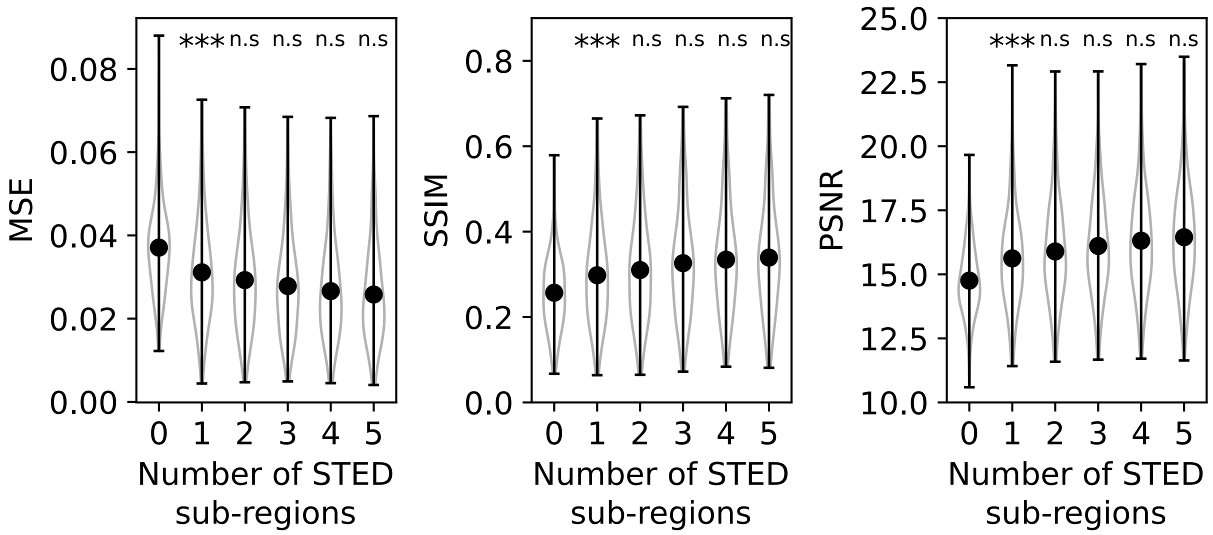

Supplementary Figure 25: Generation performance as a function of the number of STED sub-regions given as input to the generator for live-cell imaging assistance. Black dots are the distribution means. Adding one live-cell STED sub-region (100 x 100 pixels) to the input (500 x 500 pixels) of the generator at inference time significantly improves the generation accuracy in terms of MSE, SSIM, and PSNR in the central ROI (300x x 300 pixels). The generation accuracy does not further improve when using more than one STED sub-region. n=168 images. Violin plots show the minimum and maximum of each distribution, and black dots identify the mean. Statistical analysis: two-sided Mann-Whitney U-Test for the null hypothesis that the distribution underlying the values for n sub-regions is different to the distribution underlying the values for $n-1$ sub-regions. $***$ $p < 0.001$, $**$ $p < 0.01$, n.s $p > 0.05$. MSE: $p = 5 \times 10^{-7}$, 0.14, 0.29, 0.30, and 0.48 for 1, 2, 3, 4 and 5 regions respectively. SSIM: $p =$0.0013, 0.34, 0.26, 0.54, and 0.69 for 1, 2, 3, 4 and 5 regions respectively. PSNR: $p = 3 \times 10^{-6}$, 0.21, 0.27, 0.38, and 0.50 for 1, 2, 3, 4 and 5 regions respectively.

References

[1] Lavoie-Cardinal, F., Bilodeau, A., Lemieux, M., Gardner, M., Wiesner, T., Laramée, G., Gagné, C. & De Koninck, P. Neuronal activity remodels the F-actin based submembrane lattice in dendrites but not axons of hippocampal neurons. *Scientific Reports*. **10**, 11960 (2020,7), https://www.nature.com/articles/s41598-020-68180-2, Number: 1 Publisher: Nature Publishing Group

[2] He, K., Zhang, X., Ren, S. & Sun, J. Deep Residual Learning for Image Recognition. *IEEE Conference On Computer Vision And Pattern Recognition*. pp. 770-778 (2016)

[3] Ronneberger, O., Fischer, P. & Brox, T. U-Net: Convolutional Networks for Biomedical Image Segmentation. (arXiv,2015,5), http://arxiv.org/abs/1505.04597, arXiv:1505.04597

[4] Li, C. & Wand, M. Precomputed Real-Time Texture Synthesis with Markovian Generative Adversarial Networks. (arXiv,2016,4), http://arxiv.org/abs/1604.04382, arXiv:1604.04382

[5] Lehtinen, J., Munkberg, J., Hasselgren, J., Laine, S., Karras, T., Aittala, M. & Aila, T. Noise2Noise: Learning Image Restoration without Clean Data. (arXiv,2018,10), http://arxiv.org/abs/1803.04189

[6] Zhang, K., Zuo, W., Chen, Y., Meng, D. & Zhang, L. Beyond a Gaussian Denoiser: Residual Learning of Deep CNN for Image Denoising. *IEEE Transactions On Image Processing*. **26**, 3142-3155 (2017,7), http://arxiv.org/abs/1608.03981,arXiv:1608.03981

[7] Zhang, Y., Zhu, Y., Nichols, E., Wang, Q., Zhang, S., Smith, C. & Howard, S. A Poisson-Gaussian Denoising Dataset with Real Fluorescence Microscopy Images. (arXiv,2019,4), http://arxiv.org/abs/1812.10366

[8] Weigert, M., Schmidt, U., Boothe, T., Müller, A., Dibrov, A., Jain, A., Wilhelm, B., Schmidt, D., Broaddus, C., Culley, S., Rocha-Martins, M., Segovia-Miranda, F., Norden, C., Henriques, R., Zerial, M., Solimena, M., Rink, J., Tomancak, P., Royer, L., Jug, F. & Myers, E. Content-aware image restoration: pushing the limits of fluorescence microscopy. *Nature Methods*. **15**, 1090-1097 (2018,12), https://www.nature.com/articles/s41592-018-0216-7, Number: 12 Publisher: Nature Publishing Group

[9] Chen, J., Sasaki, H., Lai, H., Su, Y., Liu, J., Wu, Y., Zhovmer, A., Combs, C., Rey-Suarez, I., Chang, H., Huang, C., Li, X., Guo, M., Nizambad, S., Upadhyaya, A., Lee, S., Lucas, L. & Shroff, H. Three-dimensional residual channel attention networks denoise and sharpen fluorescence microscopy image volumes. *Nature Methods*. **18**, 678-687 (2021,6), https://www.nature.com/articles/s41592-021-01155-x, Number: 6 Publisher: Nature Publishing Group

[10] Wang, X., Yu, K., Wu, S., Gu, J., Liu, Y., Dong, C., Loy, C., Qiao, Y. & Tang, X. ESRGAN: Enhanced Super-Resolution Generative Adversarial Networks. (arXiv,2018,9), http://arxiv.org/abs/1809.00219,arXiv:1809.00219

[11] Wang, X., Xie, L., Dong, C. & Shan, Y. Real-ESRGAN: Training Real-World Blind Super-Resolution with Pure Synthetic Data. (arXiv,2021,8), http://arxiv.org/abs/2107.10833,arXiv:2107.10833

[12] Isola, P., Zhu, J., Zhou, T. & Efros, A. Image-to-Image Translation with Conditional Adversarial Networks. (arXiv,2018,11), http://arxiv.org/abs/1611.07004, arXiv:1611.07004

[13] Xu, K., Zhong, G. & Zhuang, X. Actin, spectrin, and associated proteins form a periodic cytoskeletal structure in axons. *Science*. **339**, 452-456 (2013)

[14] Mann, H. B. & Whitney, D. R. On a test of whether one of two random variables is stochastically larger than the other. *The annals of mathematical statistics* 50–60 (1947)

[15] Lagache, T. et al. Mapping molecular assemblies with fluorescence microscopy and object-based spatial statistics. *Nature Communications* 9, 1–15 (2018)

[16] Spahn, C., Mariscal, E., Laine, R., Pereira, P., Chamier, L., Conduit, M., Pinho, M., Jacquemet, G., Holden, S., Heilemann, M. & Henriques, R. DeepBacs for multi-task bacterial image analysis using open-source deep learning approaches. *Communications Biology*. **5**, 688 (2022,7)

[17] Bradski, G. The OpenCV Library. *Dr. Dobb's Journal Of Software Tools*. (2000)

[18] Wiesner, T., Bilodeau, A., Bernatchez, R., Deschênes, A., Raulier, B., De Koninck, P. & Lavoie-Cardinal, F. Activity-Dependent Remodeling of Synaptic Protein Organization Revealed by High Throughput Analysis of STED Nanoscopy Images. *Frontiers In Neural Circuits*. **14** (2020), https://www.frontiersin.org/articles/10.3389/fncir.2020.00057
