# Supplementary material for: Resolution enhancement with a task-assisted GAN to guide optical nanoscopy image analysis and acquisition
Source: Nat Mach Intell. 2023 Jul 27;5(8):830–44. doi: 10.1038/s42256-023-00689-3 (PMC10442226; doi:10.1038/s42256-023-00689-3)
Supplement: Supplementary file 2 — Reporting Summary [file 42256_2023_689_MOESM2_ESM.pdf]

nature portfolio

Corresponding author(s): Flavie Lavoie-Cardinal

Last updated by author(s): May 11, 2023

Reporting Summary

Nature Portfolio wishes to improve the reproducibility of the work that we publish. This form provides structure for consistency and transparency in reporting. For further information on Nature Portfolio policies, see our Editorial Policies and the Editorial Policy Checklist.

Statistics

For all statistical analyses, confirm that the following items are present in the figure legend, table legend, main text, or Methods section.

n/a	Confirmed	
☐	☒	The exact sample size (*n*) for each experimental group/condition, given as a discrete number and unit of measurement
☐	☒	A statement on whether measurements were taken from distinct samples or whether the same sample was measured repeatedly
☐	☒	The statistical test(s) used AND whether they are one- or two-sided<br>*Only common tests should be described solely by name; describe more complex techniques in the Methods section.*
☒	☐	A description of all covariates tested
☒	☐	A description of any assumptions or corrections, such as tests of normality and adjustment for multiple comparisons
☐	☒	A full description of the statistical parameters including central tendency (e.g. means) or other basic estimates (e.g. regression coefficient) AND variation (e.g. standard deviation) or associated estimates of uncertainty (e.g. confidence intervals)
☐	☒	For null hypothesis testing, the test statistic (e.g. *F*, *t*, *r*) with confidence intervals, effect sizes, degrees of freedom and *P* value noted<br>*Give P values as exact values whenever suitable.*
☒	☐	For Bayesian analysis, information on the choice of priors and Markov chain Monte Carlo settings
☒	☐	For hierarchical and complex designs, identification of the appropriate level for tests and full reporting of outcomes
☒	☐	Estimates of effect sizes (e.g. Cohen's *d*, Pearson's *r*), indicating how they were calculated

Our web collection on statistics for biologists contains articles on many of the points above.

Software and code

Policy information about availability of computer code

Data collection	Confocal and STED images were acquired on a 4 color Abberior Expert-Line STED microscope (Abberior Instruments GmbH, Germany), equipped with a 100x 1.4 NA oil objective and using pulsed (40 MHz) excitation (640 nm) and depletion (775 nm) lasers. Pixel size was set to 20 nm for the Axonal F-actin dataset, the Dendritic F-actin dataset and the Live F-actin dataset for both confocal and STED images. For the Synaptic protein dataset, pixel size was set to 15 nm for the STED images and 60 nm for the confocal images.
Data analysis	OpenCV 3.1.0 was used for image registration. Flask 2.0.3 was used for communicating between the microscope computer and the GPU-equipped computer. Specpy 1.2.1 was used to automate the acquisitions on the Abberior STED microscope. For training and testing TA-GAN, we used Python 3.6 with the package versions mentioned in the project Dockerfile (https://github.com/FLClab/TA-GAN/blob/main/TAGAN-Docker/Dockerfile): pytorch (1.0.0), torchvsion (0.2.1), numpy (1.19.2), Pillow (8.3.1), tifffile (2020.0.3), scipy (1.5.4), and scikit-image (0.17.2).

For manuscripts utilizing custom algorithms or software that are central to the research but not yet described in published literature, software must be made available to editors and reviewers. We strongly encourage code deposition in a community repository (e.g. GitHub). See the Nature Portfolio guidelines for submitting code & software for further information.

Data

Policy information about availability of data

All manuscripts must include a data availability statement. This statement should provide the following information, where applicable:

- Accession codes, unique identifiers, or web links for publicly available datasets
- A description of any restrictions on data availability
- For clinical datasets or third party data, please ensure that the statement adheres to our policy

The datasets used to train and test the TA-GAN model and the baselines are available for download at https://s3.valeria.science/flclab-tagan/index.html. The publicly available S. aureus dataset from Spahn et al. 2022 is available at https://zenodo.org/record/5550933#.Y6IhFNLMJH4 and https://zenodo.org/record/5551141#.Y6IjBdLMJH5.
Sample test images are available at https://github.com/FLClab/TA-GAN in the "test" subfolders of each dataset. Results from the paper can be reproduced with the complete test sets, all available to download at https://s3.valeria.science/flclab-tagan/index.html (10.5281/zenodo.7908914).

Human research participants

Policy information about studies involving human research participants and Sex and Gender in Research.

Reporting on sex and gender	N/A
Population characteristics	N/A
Recruitment	N/A
Ethics oversight	N/A

Note that full information on the approval of the study protocol must also be provided in the manuscript.

Field-specific reporting

Please select the one below that is the best fit for your research. If you are not sure, read the appropriate sections before making your selection.

☒ Life sciences ☐ Behavioural & social sciences ☐ Ecological, evolutionary & environmental sciences

For a reference copy of the document with all sections, see nature.com/documents/nr-reporting-summary-flat.pdf

Life sciences study design

All studies must disclose on these points even when the disclosure is negative.

Sample size	Training of the TA-GAN on already existing and published datasets was performed using all available images. The size of these public datasets determined the sample size for each experiment. This includes the Axonal F-actin dataset, Dendritic F-actin dataset, Synaptic protein dataset, and the DeepBacs dataset. For the live F-actin dataset, no sample size calculation was performed for the acquisition of the dataset. To estimate the number of images required to train the TA-CycleGAN, the network was trained on partial datasets and its performance was evaluated on a validation dataset. New images were acquired until the synthetic STED images generated from the validation confocal images could not be told apart from real STED images by experts. The live F-actin training dataset consists of 753 paired confocal and STED images of F-actin structures of 168 neurons from 6 independent neuronal cell culture preparation. The performance of the TA-GAN on images from each batch was compared and no difference between the batches was measured.
Data exclusions	Axonal F-actin dataset : we excluded 31 images containing mostly background, where no structures had been annotated.<br>Dendritic F-actin dataset : All images from the original dataset were included in this study.<br>Synaptic protein dataset : All images for three pairs of proteins (PSD95-Bassoon, PSD95-Homer1c and Bassoon-Homer1c) from the original dataset were included in this study.<br>Live F-actin dataset : Some images were discarded based on visual inspection (loss of focus, sample drift, imaging artifacts).The imaging sequences included in the manuscript were selected to best illustrate specific use cases of the approach (e.g. unexpected biological change, expected biological change). Other imaging sequences were included in the supplementary materials.
Replication	Time-lapse STED imaging using the TA-GAN assistance were replicated on 53 neurons from 5 independent primary neuronal cultures. On each coverslip, the experiment was performed on 3 regions from the same neuron.<br><br>Once optimal hyperparameters had been selected from multiple trials, all trainings of the neural networks were replicated twice with different initial randomization of the initial weights, using the same train/valid splits and the same hyperparameters, to verify convergence and ensure reproducibility of the results. All results from the paper are produced from the first trained models, with the second trained model only used for validation of the reproducibility. Time-lapse STED imaging using the TA-GAN assistance were replicated on 53 neurons from 5 independent primary neuronal cultures. On each coverslip, the experiment was performed on 3 regions from the same neuron.

	Once optimal hyperparameters had been selected from multiple trials, all trainings of the neural networks were replicated twice with different initial randomization of the initial weights, using the same train/valid splits and the same hyperparameters, to verify convergence and ensure reproducibility of the results. All results from the paper are produced from the first trained models, with the second trained model only used for validation of the reproducibility.
Randomization	The train/valid splits of each dataset are random. For the datasets taken from previous publications, the same test sets were used so that results could be compared.
Blinding	All user-studies were blind: the generation approach as well as the name and acquisition parameters of the images were not displayed to the participant, and the participant was not aware of whether the image was real or generated.<br><br>For all experiments performed on already acquired datasets, blinding was not relevant as all images were associated to the same experimental group and training/validation set split was done randomly, while keeping the same test set as in the original publication.<br><br>Blinding was not required for the acquisition of the live F-actin dataset as images were acquired from samples belonging to a single experimental group. Split of the live F-actin dataset in training/validation/test sets was done randomly.<br><br>For the acquisition of the TA-GAN assisted imaging sequences, investigators were blinded during the region selection process concerning which of 3 approaches would be used on a specific region: 1) TA-GAN assisted imaging, 2) control sequences of paired STED and confocal images, 3) only STED acquisitions.

Reporting for specific materials, systems and methods

We require information from authors about some types of materials, experimental systems and methods used in many studies. Here, indicate whether each material, system or method listed is relevant to your study. If you are not sure if a list item applies to your research, read the appropriate section before selecting a response.

Materials & experimental systems

n/a	Involved in the study	
☐	☒	Antibodies
☒	☐	Eukaryotic cell lines
☒	☐	Palaeontology and archaeology
☐	☒	Animals and other organisms
☒	☐	Clinical data
☒	☐	Dual use research of concern

Methods

n/a	Involved in the study	
☒	☐	ChIP-seq
☒	☐	Flow cytometry
☒	☐	MRI-based neuroimaging

Antibodies

Antibodies used

Axonal F-actin dataset :
Mouse-anti-SMI31, Biolegend, cat. 8016011:250, https://www.biolegend.com/it-it/products/purified-anti-neurofilament-h-nf-h-phosphorylated-antibody-11476
Goat anti mouse STAR580, Abberior 2-0002-005-1, Dilution 1:250, https://abberior.shop/abberior-STAR-580-goat-anti-mouse-IgG-500-ll-1-mg-ml
Phalloidin-STAR635, Abberior, cat. 2-0205-002-5, Dilution 1:50, https://abberior.shop/abberior-STAR-635-phalloidin-20-lg

Dendritic F-actin dataset :
Rabbit-anti-MAP2, Milipore Sigma, cat. AB5622, Dilution 1:1000, https://www.sigmaaldrich.com/CA/en/product/mm/ab5622
Goat anti Rabbit-STAR488, Abberior 2-0012-006-5, Dilution 1:250, https://abberior.shop/abberior-STAR-488-goat-anti-rabbit-IgG-500-ll-1-mg-ml
Phalloidin-STAR635, Abberior, cat. 2-0205-002-5, Dilution 1:50, https://abberior.shop/abberior-STAR-635-phalloidin-20-lg

Synaptic protein dataset :
Primary antibodies :
Mouse anti-PSD95 (6G6-1C9), Abcam MA1-045, Dilution 1:500, https://www.thermofisher.com/antibody/product/PSD-95-Antibody-clone-6G6-1C9-Monoclonal/MA1-045
Rabbit anti-Bassoon, Synaptic Systems 141003, Dilution 1:500, https://sysy.com/product/141003
Rabbit anti-Homer1c, Synaptic Systems 160023, Dilution 1:500, https://sysy.com/product/160023
Oregon Green 488 Phalloidin, ThermoFisher O7466, Dilution 1 : 50
https://www.thermofisher.com/order/catalog/product/O7466?SID=srch-hj-O7466
Goat anti Mouse STAR 635P, Abberior 2-0002-007-5, Dilution 1 : 250, https://abberior.shop/abberior-STAR-635P-goat-anti-mouse-IgG-500-ll-1-mg-ml
Goat anti Mouse Alexa Fluor 594, Thermofisher A11005, Dilution 1:100, https://www.thermofisher.com/antibody/product/Goat-anti-Mouse-IgG-H-L-Cross-Adsorbed-Secondary-Antibody-Polyclonal/A-11005
Goat anti Rabbit STAR 635P, Abberior 2-0012-007-5, Dilution 1 : 250, https://abberior.shop/abberior-STAR-635P-goat-anti-rabbit-IgG-500-ll-1-mg-ml
Goat anti Rabbit Alexa Fluor 594, Thermofisher A11037, Dilution 1:100, https://www.thermofisher.com/antibody/product/Goat-anti-Rabbit-IgG-H-L-Highly-Cross-Adsorbed-Secondary-Antibody-Polyclonal/A-11037

	Live F-actin dataset :<br>SiR-Actin, Supplier: Cytoskeleton inc., Cat: CY-SC001, Manufacturer's website : https://www.cytoskeleton.com/sir-actin.
Validation	Axonal F-actin dataset and Dendritic F-actin dataset : Lavoie-Cardinal, F. et al. Neuronal activity remodels the F-actin based submembrane lattice in dendrites but not axons of hippocampal neurons. Scientic reports 10, 1{17 (2020).<br>Synaptic protein dataset : Wiesner, T. et al. Activity-dependent remodeling of synaptic protein organization revealed by high throughput analysis of STED nanoscopy images. Frontiers in neural circuits 14 (2020).<br>Live F-actin dataset : Manufacturer's website : https://www.cytoskeleton.com/sir-actin

Animals and other research organisms

Policy information about studies involving animals; ARRIVE guidelines recommended for reporting animal research, and Sex and Gender in Research

Laboratory animals	Neuronal cultures were prepared from neomatal Sprague Dawley rats (P0).
Wild animals	The study did not involve wild animals.
Reporting on sex	Sex was not considered in this study.
Field-collected samples	The study did not involve samples collected from the field.
Ethics oversight	All procedures were approved by the animal care committee of Université Laval.

Note that full information on the approval of the study protocol must also be provided in the manuscript.
